# Reproducible Interferon-Centered Microglial Response at 7 Days After Traumatic Brain Injury: A Transcriptomic Meta-Analysis with Focus on the BTK/NOX2 Panel

**DOI:** 10.3390/biology15211915

**Published:** 2026-10-22

**Authors:** Yousif Salah, Ahmed Nadeem, Faleh Alqahtani

**Affiliations:** Department of Pharmacology and Toxicology, College of Pharmacy, King Saud University, Riyadh 11451, Saudi Arabia; ymohamedali@ksu.edu.sa (Y.S.); anadeem@ksu.edu.sa (A.N.)

**Keywords:** traumatic brain injury, microglia, meta-analysis, RNA-seq, neuroinflammation, oxidative stress, BTK, NOX2, Interferons

## Abstract

Traumatic brain injury (TBI) drives sustained microglial activation and secondary neurodegeneration. The Bruton’s tyrosine kinase (BTK)–NADPH oxidase 2 (NOX2) axis has been proposed as a convergent node, but transcriptional evidence has largely been derived from individual studies. Our primary aim was to characterize the microglial response at 7 days post-injury, and our secondary aim was to evaluate BTK/NOX2-associated genes within this response. We performed a random-effects meta-analysis (metafor, REML) of four independent mouse microglial RNA-seq datasets (16,636 genes). Differential expression estimates were pooled across datasets and characterized by protein–protein interaction network analysis, functional enrichment (GO, Reactome, KEGG), weighted gene co-expression analysis, and gene set enrichment analysis. A curated 14-gene BTK/NOX2 panel was tested at the individual-gene and aggregate levels. The meta-analysis identified 160 significant genes at 7 days (117 upregulated, 43 downregulated). Interferon signaling was the most consistent program, supported by gene set enrichment, over-representation analysis, and the largest network cluster. It comprised antigen presentation, a lipid-handling signature resembling disease-associated microglia (*Lpl*, log_2_FC = 3.86), and partially reduced homeostatic genes. Within the panel, *Cybb* was the only significant gene (log_2_FC = 1.66, adjusted *p* = 0.019) that increased in the same direction in all four datasets, whereas no significant change was detected at the panel level, although statistical power was limited by the small number of genes (*n* = 14). The 7-day microglial response is interferon-centered and reproducible, and the BTK/NOX2 signal is limited to a transcript-level increase in *Cybb*.

## Simple Summary

Traumatic brain injury causes lasting activation of microglia, the brain’s resident immune cells, which may involve the Bruton’s tyrosine kinase (BTK) and NADPH oxidase 2 (NOX2) pathway. We combined four independent mouse microglia studies at seven days after injury to identify genes that reproducibly change across models. This meta-analysis identified 160 significantly changed genes. Interferon signaling, an immune response normally directed against infection, was the most consistent feature, and it was accompanied by increased expression of genes for antigen presentation and lipid handling and a partial reduction in genes that characterize resting microglia. Among the 14 genes associated with BTK and NOX2, only *Cybb*, which encodes a component of the NOX2 enzyme, showed a significant increase in the same direction in all four studies. No significant changes were detected across the panel as a whole, although the panel-level analyses had limited statistical power. Together, these findings demonstrate a reproducible microglial response seven days after brain injury and identify *Cybb* as a candidate gene for further investigation of the interaction between BTK and NOX2.

## 1. Introduction

Traumatic brain injury (TBI) is a major global health problem, affecting an estimated 69 million people worldwide each year [1]. In the United States alone, approximately 1.7 million new cases occur annually, and an estimated 5.3 million people live with TBI-related disability [2]. The pathophysiology of TBI involves both primary and secondary injury mechanisms. Whereas the primary injury results from the immediate mechanical damage sustained at the time of impact, secondary injury develops over the subsequent hours to weeks through processes such as oxidative stress, excitotoxicity, blood–brain barrier (BBB) breakdown, and sustained neuroinflammation [3,4]. Because these secondary processes continue to evolve after the initial trauma, they represent potentially modifiable therapeutic targets. Among them, neuroinflammation is particularly prolonged and heterogeneous, involving CNS-resident and infiltrating immune cells across a wide signaling landscape. However, clinical trials involving anti-inflammatory agents have not consistently improved outcomes, highlighting the need for more targeted, cell-specific approaches [5]. Oxidative stress and inflammation may further amplify one another, as reactive oxygen species (ROS) promote BBB disruption and activate inflammatory cascades, while activated immune cells generate additional ROS [6]. This oxidative-inflammatory cycle can further compromise the BBB, permitting peripheral immune cell entry [7].

Microglia are the principal immune responders in this process [8,9]. Transcriptomic studies have increasingly moved beyond the traditional M1/M2 classification toward the disease-associated microglia (DAM) framework, which is characterized by a coordinated loss of homeostatic gene expression and increased expression of injury-response genes [10]. The findings of the present study are therefore interpreted within this framework. Despite this conceptual progress, individual TBI microglia transcriptomic studies remain limited by small sample sizes and model-specific variation in injury type, tissue sampling, and isolation method, making any single-dataset signal difficult to generalize. Single-dataset studies and single-cell atlases have reported a type I interferon response [11] and DAM-like states [12] in microglia after TBI, but it remains unclear whether these programs are reproducible across injury models and laboratories. A random-effects meta-analysis across independent datasets is better suited to this problem, since it explicitly quantifies between-study heterogeneity (*I*^2^, τ^2^) rather than treating one dataset as representative of the field. Therefore, this study aimed to characterize the microglial response at 7 days post-injury (dpi) through a comprehensive, multi-layer transcriptomic meta-analysis of four independent datasets representing this subacute timepoint.

As a secondary aim, this study examined the BTK/NOX2 axis as a candidate pathway within this activation state. Bruton’s tyrosine kinase (BTK) is a Tec-family kinase expressed in myeloid cells and microglia [13] and functions downstream of Toll-like receptors, Fc receptors, and NLRP3 (NOD-, LRR- and pyrin domain-containing protein 3) inflammasome signaling [14,15]. BTK inhibition has shown beneficial effects in several CNS injury models. In TBI, inhibition of Met/ hepatocyte growth factor receptor (HGFR) signaling upstream of BTK reduced BTK phosphorylation and improved neuronal survival [16], while beneficial effects have also been reported in multiple sclerosis, spinal cord injury, chronic cerebral hypoperfusion, and ischemic stroke [14,17,18,19,20]. Nicotinamide adenine dinucleotide phosphate (NADPH) oxidase 2 (NOX2) is a major source of injury-associated ROS in microglia. The enzyme complex is composed of membrane-bound subunits, including CYBB/gp91phox and CYBA/p22phox, together with cytosolic subunits, and has been recognized as a potential therapeutic target in TBI [21,22,23]. However, the relationship between BTK and NOX2 remains unclear. BTK has been reported to suppress NOX2 activity in some myeloid contexts [24] but to promote it in others [25]. Consistent with this uncertainty, a recent review of mechanisms related to BTK inhibitor-induced hypertension concluded that direct evidence linking BTK to NADPH oxidase remains limited [26]. Because BTK activity is regulated primarily by phosphorylation rather than transcription, transcriptomic data alone cannot establish functional coupling between the two genes. Accordingly, the present analysis evaluated the BTK/NOX2 gene panel as an exploratory candidate set, testing for reproducible transcriptional changes rather than assuming pathway-level engagement.

Our group has previously demonstrated that BTK inhibition attenuates NOX2-associated oxidative stress in a sepsis-induced acute kidney injury model using direct pharmacological inhibition and biochemical readouts within a single experimental system [27]. The present study differs in scope, model, and methodology. Rather than directly testing BTK/NOX2 coupling within a single injury model, it examines whether *Cybb*, *Btk*, and the broader BTK/NOX2 gene panel show reproducible transcriptional changes across four independent TBI datasets. The analysis focuses on microglia rather than systemic or renal immune cells and uses a meta-analytic rather than an interventional approach.

Accordingly, this study had two aims: first, to characterize the microglial response at 7 dpi across four independent datasets; second, to determine whether individual genes or the aggregate 14-gene BTK/NOX2 panel show reproducible transcriptional changes. We hypothesized that core microglial activation programs would be reproducible across datasets, while coordinated BTK/NOX2 regulation was treated as an exploratory question rather than a prespecified expected outcome.

## 2. Materials and Methods

### 2.1. Dataset Selection and Inclusion Criteria

Murine transcriptomic datasets were identified by searching the NCBI Gene Expression Omnibus (GEO) [28] (https://www.ncbi.nlm.nih.gov/geo/, accessed on 31 August 2025). The search used the Boolean query (‘traumatic brain injury’) AND (mouse) AND (microglia), together with all three possible two-term combinations of these search terms to identify potentially relevant studies that might not be retrieved by the three-term query. The search was limited to GEO; other repositories including ArrayExpress and the Sequence Read Archive (SRA) were not searched.

A total of 24 publicly available TBI-related transcriptomic datasets were screened against predefined eligibility criteria. Datasets were eligible if they: (i) used experimental mouse TBI models with appropriate sham or control groups; (ii) profiled isolated or enriched microglial populations rather than bulk brain tissue; (iii) used bulk RNA-sequencing (RNA-seq) or Microarray as the molecular profiling platform; (iv) included at least three biological replicates per group; and (v) included samples collected at 7 dpi. Datasets were excluded if they profiled non-microglial populations, used transgenic disease models other than reporter lines employed solely for cell isolation, used incompatible platforms (e.g., single-cell RNA-seq, proteomics), or combined TBI with additional pharmacological or immunological interventions.

Based on these criteria, four independent datasets were included: GSE276647, GSE283560, GSE253476, and GSE167459 [11,29,30,31]. For GSE283560, only the FACS-sorted resident microglial population (CD11b+, CD45^mid/low^, tdTomato−) was included in the present analysis. The tdTomato+ monocyte-derived macrophage population reported in the original study was excluded from the present analysis. For GSE276647, only the untreated TBI and sham or control groups were analyzed. Samples from anti-CD3 monoclonal antibody treatment arms available in the original study were not included, consistent with the predefined eligibility criteria. Full screening records for all 24 datasets assessed, including accession numbers and reasons for exclusion, are provided in Supplementary Table S1.

**Figure 1:**
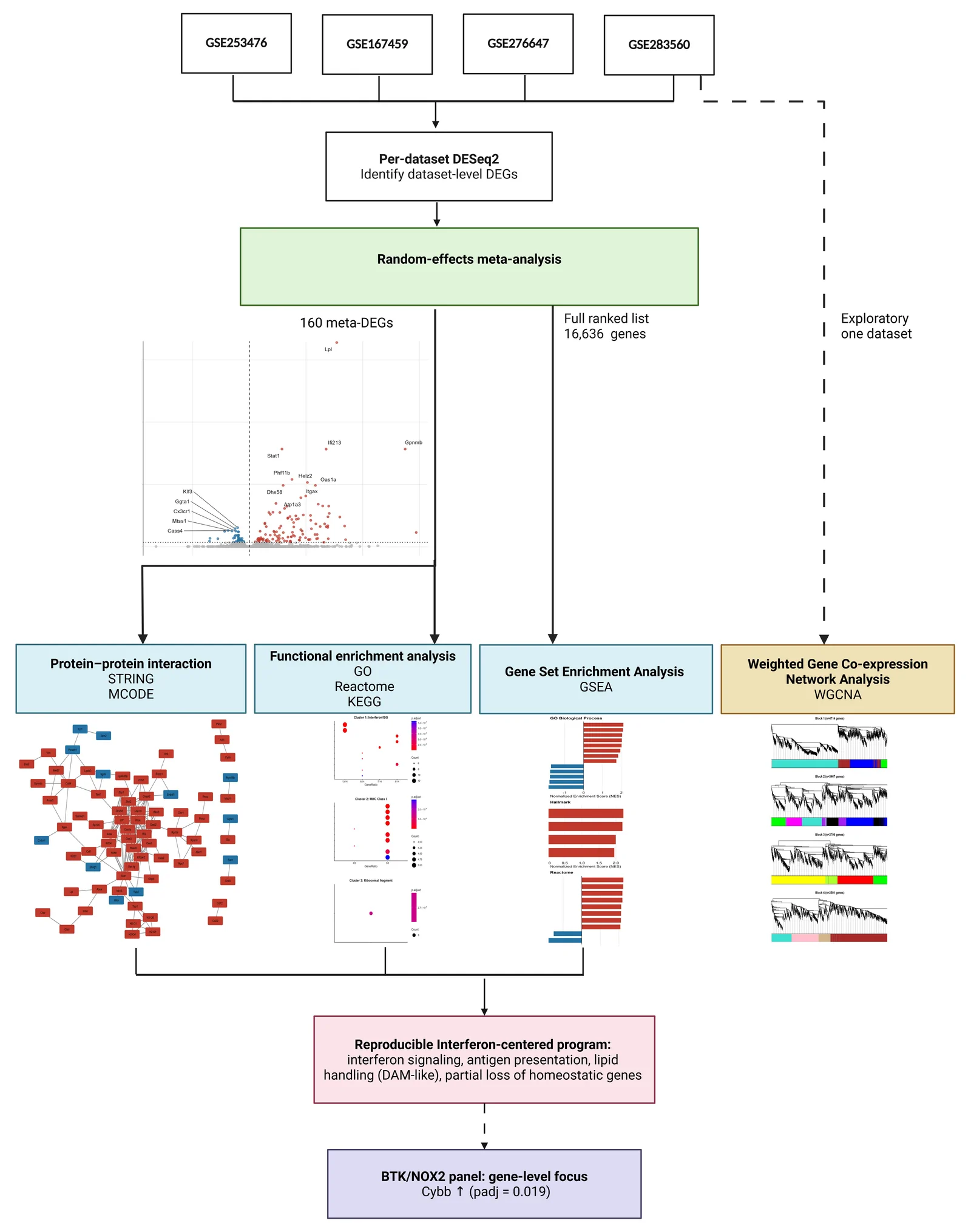
Schematic overview of the complete analytical pipeline, from dataset retrieval through six processing steps (per-dataset DESeq2 analysis, random-effects meta-analysis, PPI network analysis, functional enrichment, GSEA, and WGCNA) to two integrated findings: the reproducible genome-wide activation program and the BTK/NOX2 panel result. Box colors mark the stage: white, input and per-dataset analysis; green, meta-analysis; blue, downstream analyses; yellow, exploratory analysis; pink, integrated program; purple, gene-level focus. Solid arrows show the main analysis flow; dashed arrows show the exploratory WGCNA step (one dataset) and the gene-level follow-up. The small plots are schematic illustrations of each method; the data are in Figures 2–7. ↑, increased in TBI; *p*adj, BH-adjusted *p* value. Created with BioRender.com (VS2ACIEIPV: dated 20 October 2026).

A summary of dataset characteristics is provided in Table 1. Additional experimental parameters, including injury severity, anesthesia protocol, craniotomy dimensions, and exact age ranges, are provided in Supplementary Table S2. The overall analytical workflow is shown schematically in Figure 1. This study re-analyzed primary sequencing data from independent cohorts using a uniform analytical pipeline rather than synthesizing previously published summary statistics. Therefore, PRISMA risk-of-bias and publication-bias assessment tools, which are designed for literature-based evidence synthesis, were not considered applicable to this study design.

### 2.2. Per-Dataset Differential Expression Analysis

Each dataset was analyzed separately using a common DESeq2 differential-expression framework while retaining specified dataset-specific preprocessing decisions. Differential expression was modeled using DESeq2 1.48.1 [32] with injury status as the sole model term and the TBI group compared with the corresponding sham or control group. Full gene-level summary statistics (log_2_ fold change [log_2_FC], standard error, Wald statistic, nominal *p*-value, Benjamini–Hochberg [BH]-adjusted *p*-value) were exported for all analyzable genes without pre-filtering by significance.

#### 2.2.1. Dataset-Specific Preprocessing

**GSE276647**. This dataset’s input count matrix was obtained as a pre-processed GEO Supplementary File with mouse gene symbols as row identifiers, whereas the other three datasets used ENSEMBL identifiers. These gene-level counts were rounded to integers and used as DESeq2 input, and gene identifiers were mapped via AnnotationDbi::select() 1.70.0 [33] using keytype = ‘ALIAS’ to resolve symbol synonyms against org.Mm.eg.db [34]. After low-count filtering, 14,901 of 15,718 genes were retained. Log_2_FC estimates were regularized using the apeglm 1.30.0 shrinkage estimator [35].

**GSE283560**. Transcript-level quantification was performed by the original authors with Salmon (nf-core/rnaseq, 3.12.0) and imported via tximport 1.36.1 [36], then summarized to gene-level counts for DESeq2. Transcript-to-gene mapping used the Ensembl GRCm39 (release 110) annotation. Raw counts were subset to the 11 samples collected at 7 dpi prior to normalization, ensuring size factors were estimated from the subset rather than a larger multi-timepoint sample pool. Each sample was sequenced across three technical-replicate lanes, and counts were summed across all three lanes per sample prior to normalization. Low-count genes were removed using a total-count filter, retaining genes with rowSums(counts) ≥ 10 (46,201 genes). Gene symbols were mapped from ENSEMBL IDs via org.Mm.eg.db. During duplicate-symbol resolution, 40 rows were removed while retaining the row with the higher baseMean for each duplicated symbol (46,161 genes retained). Log_2_FC estimates were regularized using the apeglm shrinkage estimator [35].

**GSE253476**. Fractional scaled Salmon gene counts (ENSEMBL IDs) were rounded to integers for DESeq2 input. Low-count genes were removed using a total-count filter, retaining genes with rowSums(counts) ≥ 10 (19,016 of 45,706 genes). Log_2_FC estimates were regularized using the apeglm shrinkage estimator [35].

**GSE167459**. Raw gene-level counts (ENSEMBL IDs) were used as DESeq2 input. GEO lists ten microglia samples for this series (five Sham, five FPI). The primary analysis used six of them (three Sham, three FPI), selected by sample code pattern M_S in the count file. These six formed one cluster and were analyzed without a batch term. The other four (308J-A, 309J-A, 310J-A, 315J-A) formed a separate batch on the second principal component. For this reason, the analysis with all ten samples, with and without batch in the model, is reported as a sensitivity analysis (Table S18; Figure S9). Low-count genes were removed using a total-count filter, retaining genes with rowSums(counts) ≥ 10 (16,211 genes). Log_2_FC estimates were regularized using the apeglm shrinkage estimator [35].

#### 2.2.2. Shared Preprocessing Parameters

Low-count genes were removed prior to testing using a consistent total-count filter across all four datasets while retaining genes with rowSums(counts) ≥ 10. Apeglm shrinkage [35] was applied uniformly across all four datasets as the primary analysis design. Two datasets (GSE253476, GSE167459) natively use apeglm shrinkage in their own published pipelines. For the remaining two datasets (GSE276647, GSE283560), which natively report unshrunk maximum-likelihood estimates, apeglm shrinkage was applied here to achieve a harmonized effect-size treatment across all pooled studies. This harmonized design and its comparison against a fully unshrunk alternative are presented as a formal two-arm sensitivity analysis in Section 2.3.1.

#### 2.2.3. Sample-Level Quality Control

Prior to differential expression analysis, sample-level quality control was performed identically for all four datasets using three independent metrics computed from variance-stabilized counts: principal component analysis (PCA) on the top 500 most variable genes, Spearman correlation between samples, and library size distributions. A sample was flagged as a candidate outlier only when it showed clear separation on PCA together with reduced within-group correlation. Library size was used as a supporting metric where informative, since DESeq2’s size-factor normalization corrects directly for sequencing-depth differences. Therefore, a high-depth sample was not treated as equally concerning as a low-depth one. This criterion identified a clear candidate in three of the four datasets. In three datasets, one sample separated clearly from its group on PCA and had the lowest within-group correlation (GSE167459: 1304M_S77; GSE276647: TBI_Iso_1; GSE283560: GSM8666331). In GSE253476, no sample separated clearly (within-group correlation 0.924–0.934), so no sample was identified as a quality-control outlier. Sham_Mg_R3, the sample with the highest library size, was excluded only in the leave-one-sample-out analysis (Section 2.3.4), so that every dataset was tested in the same way. Full quality control figures for all four datasets are provided in Supplementary Figures S1–S4.

#### 2.2.4. Outlier Handling

DESeq2 automatically replaces individual outlier count values, identified using Cook’s distance, only when the affected sample’s group reaches a minimum replicate threshold (default minReplicatesForReplace = 7). Among the four datasets, only GSE283560’s TBI group (*n* = 8) meets this threshold. GSE276647 (largest group *n* = 4), GSE253476 (*n* = 3), and GSE167459 (*n* = 3) fall below it; therefore, the default (inactive) outlier replacement was applied to these three datasets.

For GSE283560, automatic outlier replacement was disabled (minReplicatesForReplace = Inf) because of the small and unbalanced group sizes *(n* = 3 control vs. *n* = 8 TBI), so no observations were automatically replaced in the primary analysis.

#### 2.2.5. Microglial Purity Assessment

To assess microglial isolation purity, we examined variance-stabilized expression of two canonical microglial markers (*P2ry12*, *Cx3cr1*) and two peripheral immune-cell markers (*Ptprc*/Cd45, pan-leukocyte; *Cd3e*, T-cell-specific). For each dataset, marker expression was obtained from the DESeq2 object generated using the same low-count filtering criteria and model applied in the primary differential expression analysis (Section 2.2.2). This analysis was performed to evaluate potential contamination by peripheral immune cells, because several genes in the BTK/NOX2 panel are not specific to microglia and therefore require adequate sample purity for appropriate interpretation. Full results are shown in Supplementary Figure S5 and Table S3.

### 2.3. Random-Effects Meta-Analysis (REML)

**Figure 2:**
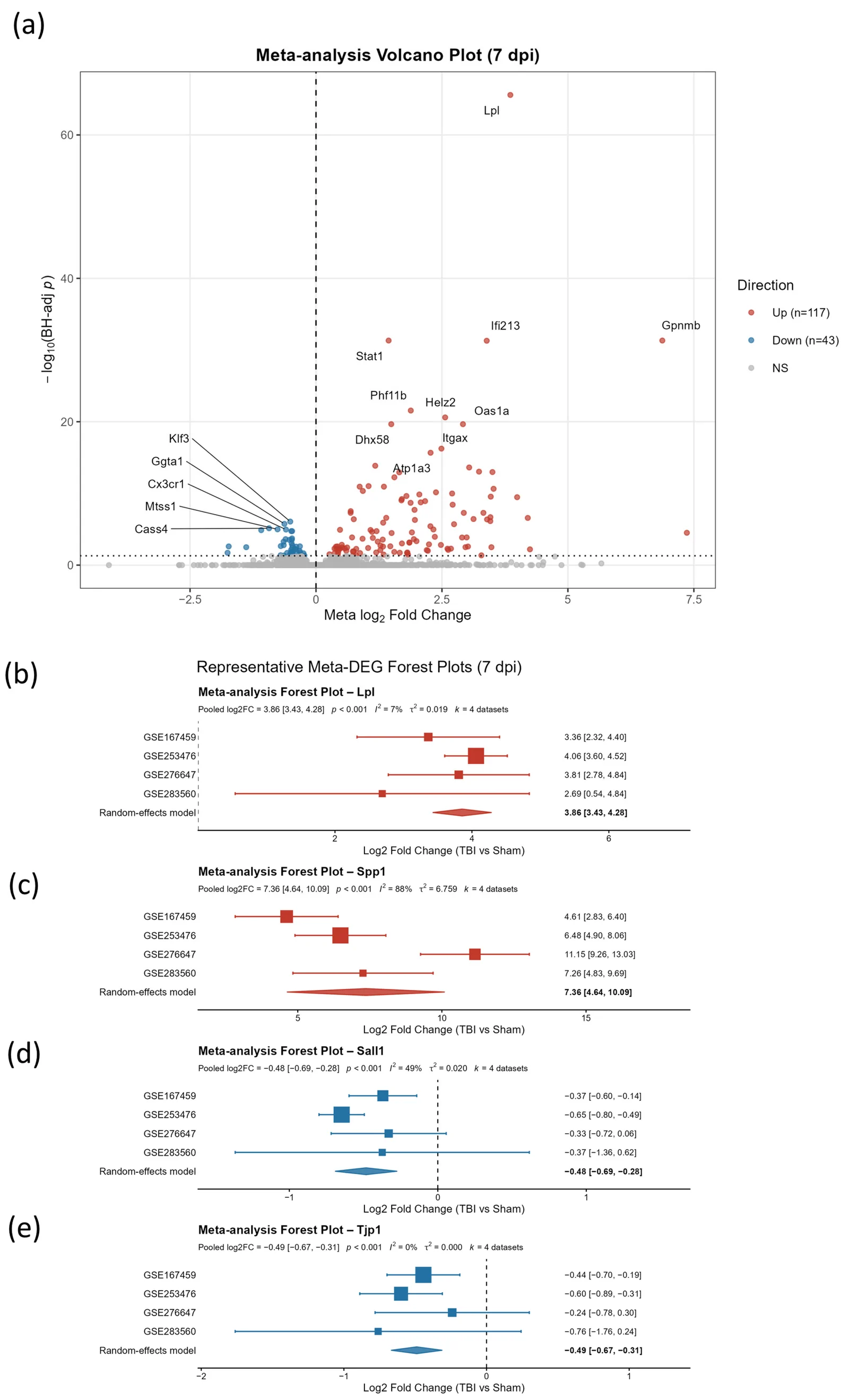
Meta-analysis results at 7 days post-TBI. (**a**) Volcano plot showing pooled log_2_ fold change versus −log10(BH-adjusted *p*-value). Red: upregulated meta-DEGs (*n* = 117); blue: downregulated meta-DEGs (*n* = 43); gray: non-significant. (**b**–**e**) Forest plots for *Lpl* (**b**), *Spp1* (**c**), *Sall1* (**d**), and *Tjp1* (**e**). In (**b**–**e**), red marks an increase and blue a decrease in TBI. Points represent individual dataset estimates; diamonds represent random-effects pooled estimates. Pooled log_2_FC [95% CI], *p*-value, *I*^2^, tau^2^, and *k* (number of datasets) are shown above each panel.

Gene-level log_2_FC estimates and corresponding standard errors from the four 7-dpi datasets were integrated using random-effects meta-analysis implemented in the metafor package 4.8-0 [37]. All four datasets were processed using the same low-count filter (rowSums ≥ 10) and shrinkage method (apeglm), ensuring consistent statistical treatment of gene-level estimates across datasets prior to meta-analytic pooling (Section 2.3.1). Analyses were restricted to genes represented in at least two datasets. Genes were matched across datasets by gene symbol. If a symbol appeared more than once in a dataset, the first row was kept (tables are sorted by adjusted *p*-value), and genes with a missing log_2_FC or lfcSE, or with lfcSE = 0, were removed before pooling. For each gene, study-level estimates were pooled using restricted maximum likelihood (REML), which estimates and accounts for between-study heterogeneity rather than assuming a single fixed true effect. For 47 genes, metafor reported that the REML algorithm did not fully converge, and the between-study variance (tau^2^) was set to 0; these genes were kept. This did not affect *Cybb*, which had tau^2^ above 0. A fixed-effects model was used as a fallback for the 2 genes for which REML did not return a result. For each gene, the pooled model returned a combined log_2_FC, 95% confidence interval, z-statistic, nominal *p*-value, and heterogeneity statistics *I*^2^ and tau^2^. Multiple-testing corrections were applied via the BH procedure. Meta-differentially expressed genes (meta-DEGs) were defined as genes with BH-adjusted *p* < 0.05, with no fold-change threshold applied. A complete meta-ranked gene list was generated using the signed statistic sign(log_2_FC) × −log10(*p*) for downstream threshold-free analyses (Section 2.7). Volcano plots were generated by plotting pooled log2 fold change against −log10(BH-adjusted *p*-value) for all analyzable genes, with meta-DEGs (*p*adj < 0.05) colored by direction. Forest plots were generated from the rma() output for individual genes, showing each dataset’s log_2_ fold change and 95% confidence interval alongside the pooled random-effects estimate (Figure 2).

#### 2.3.1. Shrinkage Sensitivity Analysis

The four datasets were originally processed under independently validated per-dataset pipelines. Two datasets natively reported apeglm-shrunk log_2_ fold-change estimates and the remaining datasets natively reported unshrunk maximum-likelihood estimates (MLE). For the primary analysis, apeglm shrinkage was applied uniformly across all four datasets to ensure that gene-level estimates were generated using the same publicly reproducible procedure before pooling (Section 2.9). We assessed the sensitivity of this choice in a secondary analysis using native MLE (fully unshrunk) estimates for all four datasets, including GSE253476 and GSE167459, which use apeglm shrinkage in their original pipelines. Full gene-level results for both analyses are provided in Supplementary Table S4, and results for the 14-gene BTK/NOX2 panel are reported in Section 3.6.

#### 2.3.2. Dataset-Representation Sensitivity Analysis

To assess whether the overall meta-analysis was influenced by genes with limited representation across studies, we repeated the analysis using only genes present in all four datasets and reapplied the BH correction within this restricted subset. The distribution of analyzable genes by number of contributing datasets is reported in Section 3.1 and Supplementary Table S5. The BTK/NOX2 panel gene results under this restriction are reported in Section 3.6.

#### 2.3.3. Leave-One-Dataset-Out Sensitivity Analysis

To test whether the genome-wide meta-analysis result depended on any single dataset, the full random-effects meta-analysis (Section 2.3) was re-run four times. Each run excluded one dataset and re-pooled the remaining three. Benjamini–Hochberg correction was re-applied within each three-dataset subset and not carried over from the four-dataset analysis. The same procedure was applied to *Cybb*, the BTK/NOX2 panel gene with elevated significance in the primary panel analysis (Section 2.8). Full results are provided in Supplementary Table S6.

#### 2.3.4. Leave-One-Sample-Out Sensitivity Analysis

For each dataset, one sample was excluded: the quality-control outlier identified in Section 2.2.3 or, for GSE253476, the sample with the highest library size. DESeq2 was then re-run for that dataset using the same dataset-specific primary analysis settings, including apeglm shrinkage and, for GSE283560, the minReplicatesForReplace = Inf setting (Section 2.2.1, Section 2.2.2, Section 2.2.3 and Section 2.2.4). The resulting sample-excluded dataset was then pooled with the three unchanged datasets using the same random-effects meta-analysis framework as in the primary analysis (Section 2.3). This procedure generated a new global meta-DEG count for each of the four leave-one-sample-out runs. The adjusted *p*-values of *Cybb*, *Fcer1g*, and *Btk* were also compared between the full and sample-excluded datasets to test whether the panel-level result depended on any single excluded sample. In each run, we also repeated the Hallmark GSEA (Section 2.7) on the new ranked list and recorded the Interferon Gamma and Interferon Alpha Response sets and the genes *Cx3cr1*, *Sall1*, and *Tjp1* (Table S7).

### 2.4. Protein–Protein Interaction Network Construction and Cluster Detection

The meta-DEGs were submitted to the STRING database [38] (v12.0) to construct a protein–protein interaction (PPI) network, retaining edges at a confidence score threshold ≥ 0.7. Network topology was visualized and analyzed in Cytoscape 3.10.4 [39] via RCy3 2.28.1 [40]. Densely connected subnetworks were identified by applying Molecular Complex Detection (MCODE) [41] to the full network using the following parameters: degree cutoff = 2, node score cutoff = 0.2, *k*-core = 2, and maximum depth = 100. To assess robustness, MCODE 2.0.3 was re-run at node score cutoffs of 0.1 and 0.3 (Supplementary Table S8). Cluster membership of the 14 curated BTK/NOX2 panel genes was recorded.

### 2.5. Functional Enrichment Analysis

Over-representation analysis (ORA) was performed separately for upregulated meta-DEGs, downregulated meta-DEGs, and each of the MCODE clusters identified in Section 2.4 (see Section 3.2). All three clusters were analyzed regardless of size because only a small number of clusters were identified. All ORA testing used a background restricted to the 16,636 analyzable genes from the meta-analysis (Section 2.3) rather than the default whole-genome background. Gene symbols were converted to ENTREZ IDs using bitr. Gene Ontology (GO) Biological Process enrichment was performed with enrichGO in clusterProfiler 4.16.0 [42], using org.Mm.eg.db 3.21.0 [34] for annotation and BH-corrected *p* < 0.05. Reactome pathway enrichment was performed with enrichPathway in ReactomePA 1.52.0 [43] using organism = ‘mouse’ and BH-corrected *p* < 0.05. Kyoto Encyclopedia of Genes and Genomes (KEGG) pathway enrichment was performed with enrichKEGG using organism = ‘mmu’ and BH-corrected *p* < 0.05. KEGG terms corresponding to viral and immune-disease pathways (e.g., ‘Coronavirus disease,’ ‘Influenza A,’ ‘Measles,’ ‘Allograft rejection’) were interpreted as reflecting shared interferon-stimulated gene (ISG) and major histocompatibility complex (MHC) membership within the KEGG database rather than evidence of disease. This annotation artifact is addressed in the Discussion. Enrichment results were interpreted separately for each gene set and cluster. Cluster-level GO Biological Process terms were simplified using clusterProfiler’s simplify() function (similarity cutoff = 0.7) to reduce semantic redundancy prior to reporting.

### 2.6. Weighted Gene Co-Expression Network Analysis (WGCNA)

WGCNA [44] was applied to GSE283560 only, because it has the largest 7-dpi sample size of the four datasets (*n* = 11; 3 control, 8 TBI, versus *n* = 6–8 for the others). Because the sample size was below the approximately 15 samples recommended by the WGCNA authors [45], the analysis was considered exploratory, and module–trait associations were not used as confirmatory evidence. Counts from the 11 samples, with technical lanes summed as described in Section 2.2.1, were filtered to retain genes with counts per million (CPM) ≥ 1 in at least three samples, yielding 15,635 genes. Gene identifiers were then mapped to gene symbols using org.Mm.eg.db [34]. After removal of unmapped genes (*n* = 2234) and resolution of duplicated symbols (*n* = 13) by retaining the transcript with the highest mean expression, 13,388 genes remained for analysis. Counts were variance-stabilizing transformed (VST, blind = TRUE; DESeq2 [32], minReplicatesForReplace = Inf) and adjusted for scaled log10 library size using limma::removeBatchEffect [46]. The model included condition (~condition) to preserve the control–TBI difference. Adjustment for technical and biological sources of variation before network inference may reduce false discoveries [47]. However, to our knowledge, this specific application of limma::removeBatchEffect for library-size adjustment in this context has not been previously reported.

A signed network was built with blockwiseModules (minModuleSize = 150, mergeCutHeight = 0.30, deepSplit = 1, maxBlockSize = 5000). The lowest soft-thresholding power achieving a signed scale-free topology fit of R^2^ ≥ 0.80 was selected [48]; if none met this criterion, a power of 18 was used [45]. Module eigengenes were correlated with TBI status (Pearson r). Because three controls among 11 samples give only 165 label arrangements, exact permutation *p*-values were computed, per module and family-wise (largest r across modules), and labels were permuted at the correlation step only. Eigengenes were also correlated with library size and a vascular/fibroblast marker score (*Col1a1*, *Col1a2*, *Pdgfrb*, *Acta2*, *Dcn*).

Robustness was tested by (i) removing GSM8666331 (flagged in Section 2.2.3 and Section 2.2.5) and repeating the pipeline on 10 samples, and (ii) leave-one-sample-out analysis (11 networks). Each run used the same minimum module size, merge height, and power selection rule, and the power was chosen again in each run. Module stability was judged as follows: a TBI-associated module was robust if family-wise *p* < 0.05 in most leave-one-out runs, fragile if only without GSM8666331, and partly stable if in some runs only. Modules were compared across runs by gene overlap since color names do not identify the same module between runs. Module membership (kME) was computed with signedKME (including the 14 panel genes, Section 2.8), and GO Biological Process enrichment (enrichGO [42], BH *p* < 0.05) was run per module against all 13,388 network genes.

### 2.7. Gene Set Enrichment Analysis (GSEA)

Gene set enrichment analysis (GSEA) was run with the fgsea package (version 1.34.2) [49]. All genes from the random-effects meta-analysis were ranked by the sign of the log_2_ fold change multiplied by the negative log10 of the nominal meta-analysis *p*-value (Section 2.3). Gene symbols were converted to ENTREZ IDs using org.Mm.eg.db [34]. When two symbols mapped to one ENTREZ ID, the one with the larger absolute rank value was kept. Gene sets came from the Molecular Signatures Database (MSigDB) [50,51] through the msigdbr package (version 25.1.1) [52]. We used human collections mapped to mouse orthologs. Three collections were tested: GO Biological Process (C5:GO:BP), Hallmark (H) [53], and Reactome (C2:CP:REACTOME). Only gene sets with 15 to 500 genes were tested. *p*-values were estimated with the fgsea multilevel method, starting from 10,000 permutations, on a single core with a fixed random seed. Gene sets with a Benjamini–Hochberg adjusted *p*-value below 0.05 were considered significant. The 14-gene BTK and NOX2 panel was tested as one gene set on the same ranked list while allowing sets of 5 to 500 genes.

### 2.8. Focused Analysis of the BTK/NOX2 Candidate Gene Panel

A curated panel of 14 genes representing the BTK and NOX2 pathways was evaluated. The NOX2 gene group comprised six genes: the membrane-bound flavocytochrome b558 subunits *Cybb* (gp91phox) and *Cyba* (p22phox), the cytosolic subunits *Ncf1* (p47phox), *Ncf2* (p67phox) and *Ncf4* (p40phox), and the small GTPase *Rac2*, which is required for activation of the complex [21,22,54]. The BTK gene group comprised eight genes selected to span the signaling cascade from immunoreceptor engagement to transcriptional output in myeloid cells: the FcRγ adaptor *Fcer1g*, which carries the Immunoreceptor tyrosine-based activation motif (ITAM) that initiates signaling [55]; the upstream kinases *Lyn* and *Syk*, which activate BTK [56]; the adaptor *Blnk* and the Src family kinase *Hck*; *Btk* itself and its substrate *Plcg2*; and the transcription factor *Nfkb1* [15,56]. *Nfkbia*, which encodes the nuclear factor kappa B inhibitor IκBα, was excluded because it restrains the pathway rather than forming part of it. The panel was defined from the literature. For each gene, the pooled log_2_FC, 95% CI (Confidence interval), *p*-value, and *I*^2^ were extracted from the genome-wide random-effects meta-analysis (Section 2.3). Significance was assessed using the Benjamini–Hochberg adjusted *p*-values from the genome-wide analysis; therefore, no additional correction was applied within the panel.

Coordinated regulation of the panel was assessed by two approaches. First, gene set enrichment analysis of the 14-gene panel was performed using the meta-ranked list (Section 2.3) and fgsea, as described in Section 2.7. The default minimum gene set size of 15 would exclude a 14-gene panel, so the minimum was set to 5 to allow the panel to be tested. This analysis served as the primary panel-level test. Second, the BTK gene group (*n* = 8) and the NOX2 gene group (*n* = 6) were tested separately by one-sample *t*-tests of the pooled log_2_FC values against zero. This was considered a secondary analysis since this test weights all genes equally and does not account for the precision of individual estimates or for correlation among co-expressed genes.

Dataset-level estimates for individual genes were shown as forest plots (Figure S6). Estimates and standard errors were taken from the DESeq2 results (Section 2.2), and 95% CIs were calculated as the estimate ± 1.96 standard errors. Study weights were obtained by refitting the random-effects model (REML) with metafor [37], and the refit reproduced the pooled estimates in the genome-wide table. *Cybb* was plotted under the harmonized analysis, and *Fcer1g* was plotted under both harmonized and unshrunk estimates (Section 2.3.1; Figure S6).

### 2.9. Statistical Software and Reproducibility

All analyses were performed in R [57] version 4.5.1 (13 June 2025) on Windows 10 x64. The following package versions were used: GEOquery 2.76.0, data.table 1.17.8, DESeq2 1.48.1, apeglm 1.30.0, tximport 1.36.1, AnnotationDbi 1.70.0, limma 3.64.3, metafor 4.8-0, WGCNA 1.73, STRINGdb 2.20.0, RCy3 2.28.1, clusterProfiler 4.16.0, org.Mm.eg.db 3.21.0, ReactomePA 1.52.0, fgsea 1.34.2, msigdbr 25.1.1, igraph 2.1.4, and ggplot2 [58] 4.0.0. Network visualization used Cytoscape [39] (v3.10.4) with the MCODE [41] plugin (v2.0.3). Figures were generated in ggplot2, with ggrepel 0.9.6 for labels and pheatmap 1.0.13 for heatmaps (exported as 300 DPI TIFF files with Arial ≥ 9 pt), and network figures were exported from Cytoscape. Figure 1 workflow schematic was created with BioRender.com (accessed on 17 October 2026). All analysis scripts (01–21), the sample-selection tables for the four datasets, and a table of parameters are available at https://github.com/Yousif-Salah/tbi-microglia-7dpi-meta-analysis (accessed on 17 October 2026) (release v1.0.0; Zenodo DOI: https://doi.org/10.5281/zenodo.23212074). During the preparation of this study, the authors used Claude Sonnet 5 (Anthropic, San Francisco, CA, USA; https://claude.ai (accessed on 17 October 2026)) to assist with R script development for the analysis pipeline. The authors reviewed, ran, and verified all code and take responsibility for the results.

## 3. Results

### 3.1. Random-Effects Meta-Analysis Reveals Significant Microglial Transcriptional Changes at 7 Days Post-TBI

To assess microglial isolation purity, we examined variance-stabilized expression of two canonical microglial markers (*P2ry12*, *Cx3cr1*) and two peripheral immune-cell markers (*Ptprc*/Cd45, *Cd3e*) across all four datasets (Figure S5, Table S3). *P2ry12* and *Cx3cr1* were elevated and consistent in all four datasets. *Cd3e* was undetectable after filtering in two datasets (GSE253476, GSE167459) and near zero in the other two. Furthermore, there was no evidence of a dataset-wide contamination signal. One sample, GSM8666331 (GSE283560, TBI), deviated from this pattern (lower *P2ry12*, higher *Cd3e*), consistent with its earlier flagging by PCA.

Across 16,636 analyzable genes, meta-analysis identified 160 meta-DEGs (117 upregulated, 43 downregulated) at BH-adjusted *p* < 0.05. Full per-gene meta-analysis statistics are provided in Table S9. The volcano plot showed a predominance of significantly upregulated genes relative to downregulated genes (Figure 2a).

*Lpl* had the smallest adjusted *p*-value in the meta-analysis (log_2_FC = 3.86, *p*adj = 2.64 × 10^−66^, *I*^2^ = 7%, *k* = 4 datasets). The pooled confidence interval is narrow (95% CI [3.43, 4.28]) despite modest per-study sample sizes (*n* = 3 to 8 per group). Consistent agreement among datasets explains this, not pooling-method artifacts. The very small *p*-value reflects the large effect size (log_2_FC = 3.86) together with the relatively small per-dataset standard errors estimated by DESeq2 for this gene, rather than the sample size alone. Other strongly upregulated genes included *Gpnmb*, *Stat1*, *Ifi213*, *Phf11b*, *Helz2*, *Oas1a*, *Dhx58*, *Itgax*, and *Atp1a3*. The most strongly downregulated genes were *Klf3*, *Ggta1*, *Cass4*, *Mtss1*, and *Cx3cr1*.

Forest plot analyses showed how individual datasets contributed to the pooled estimate for four representative genes (Figure 2b–e). *Lpl* was consistently upregulated across all four datasets, with a large pooled effect size and low between-study heterogeneity (pooled log_2_FC = 3.86, 95% CI: 3.43 to 4.28, *I*^2^ = 7%, *k* = 4). *Spp1*, a marker of disease-associated microglia, was also strongly upregulated in all four datasets, though with substantial between-study heterogeneity (pooled log_2_FC = 7.36, 95% CI: 4.64 to 10.09, *I*^2^ = 88%, *k* = 4). *Sall1* and *Tjp1*, two genes expressed in microglia, were consistently downregulated across all four datasets. Between-study heterogeneity was moderate for *Sall1* (pooled log_2_FC = −0.48, 95% CI: −0.69 to −0.28, *I*^2^ = 49%, *k* = 4) and low for *Tjp1* (pooled log_2_FC = −0.49, 95% CI: −0.67 to −0.31, *I*^2^ = 0%, *k* = 4). By contrast, canonical homeostatic microglial markers *P2ry12*, *P2ry13*, and *Gpr34* were not significant (adjusted *p* = 0.46, 0.10, and 0.12).

To test the robustness of this result, four sensitivity analyses were performed: a shrinkage sensitivity analysis, a dataset-representation analysis, a leave-one-dataset-out analysis, and a leave-one-sample-out analysis (Section 2.3.1, Section 2.3.2, Section 2.3.3 and Section 2.3.4). The fully unshrunk analysis increased the total meta-DEG count relative to the primary analysis (1411 vs. 160 genes; 915 up, 496 down). This is most plausibly explained by reintroduced small-sample estimation noise in the two lowest-replication datasets (GSE253476, GSE167459; *n* = 3 per group). *Cybb* remained significant under both treatments (primary analysis: *p*adj = 0.0188; fully unshrunk analysis: *p*adj = 0.00033). Full per-gene results across both conditions, including all 14 BTK/NOX2 panel genes, are provided in Supplementary Table S4.

Of the 16,636 analyzable genes, 2846 (17.1%) were represented in two datasets, 2567 (15.4%) in three datasets, and 11,223 (67.5%) in all four datasets. Restricting the analysis to the *k* = 4 subset and re-applying BH correction within it gave 155 meta-DEGs (109 upregulated, 46 downregulated). This result was close to the full-dataset result of 160 (117 upregulated, 43 downregulated). *Cybb*’s adjusted *p*-value was slightly stronger under this restriction (*p*adj = 0.0143) than in the full analysis (*p*adj = 0.0188).

The genome-wide leave-one-dataset-out analysis showed a global meta-DEG count ranging from 77 (GSE253476 excluded) to 311 (GSE276647 excluded), against a baseline of 160. *Cybb*’s adjusted *p*-value stayed below 0.05 in two of the four runs (GSE167459 excluded: *p*adj = 7.8 × 10^−6^; GSE276647 excluded: *p*adj = 0.025) but rose above 0.05 in the other two (GSE283560 excluded: *p*adj = 0.086; GSE253476 excluded: *p*adj = 0.700). Full results are provided in Supplementary Table S6.

A per-sample leave-one-out check was also performed. For each dataset, the selected control sample was excluded, and the dataset was re-pooled with the three unchanged datasets. The global meta-DEG count ranged from 105 (GSE283560’s flagged sample excluded) to 228 (GSE276647’s flagged sample excluded), compared with 160 in the primary analysis. The same procedure was applied to *Cybb*. Its adjusted *p*-value remained below 0.05 in three of the four runs (GSE253476 excluded: *p*adj = 0.0087; GSE276647 excluded: *p*adj = 0.0170; GSE283560 excluded: *p*adj = 0.0125), but rose above 0.05 when GSE167459’s flagged sample was excluded (*p*adj = 0.0747). In the same four analyses, the Hallmark Interferon Gamma Response (Normalized enrichment score [NES] 2.17 to 2.30; adjusted *p* ≤ 1.2 × 10^−7^) and Interferon Alpha Response (NES 2.16 to 2.26; adjusted *p* ≤ 2 ×10^−6^) stayed significantly enriched, compared with NES 2.22 and 2.19 in the full analysis. *Cx3cr1*, *Sall1* and *Tjp1* stayed lower in every run (log_2_FC −0.46 to −0.65; adjusted *p* ≤ 0.006). Full results are provided in Supplementary Table S7.

The primary analysis of GSE167459 used six of the ten microglia samples (Section 2.2.1). When all ten samples were analyzed with batch in the model, the interferon responses stayed enriched (NES 2.19 and 2.14), and *Cybb* stayed significant (BH-adjusted *p* = 7.5 × 10^−6^). Without batch in the model, *Cybb* was not significant (BH-adjusted *p* = 0.76) (Table S18; Figure S9).

### 3.2. PPI Network Analysis Identifies Functional Gene Clusters in Post-TBI Microglia

**Figure 3:**
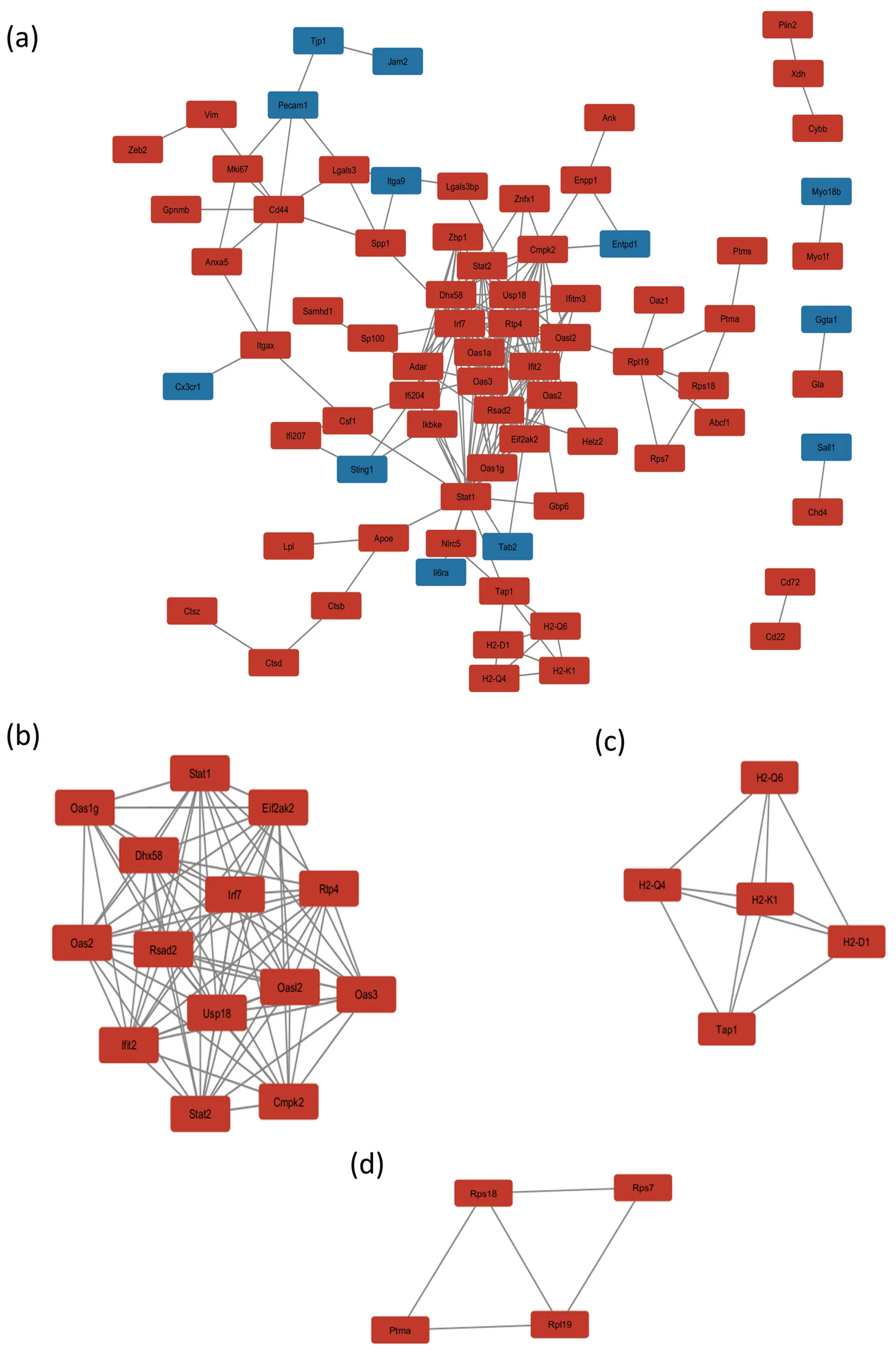
PPI network and MCODE clusters at 7 dpi. (**a**) Full STRING PPI network (confidence ≥ 0.7) of 154 meta-DEG nodes and 194 edges; red: upregulated, blue: downregulated (same colors in (**a**–**d**)). The network comprises 83 connected components: one 66-node giant component, one 3-gene component (*Plin2-Xdh-Cybb*), four 2-gene components (*Myo18b-Myo1f*, *Ggta1-Gla*, *Sall1-Chd4*, *Cd72-Cd22*), and 77 unconnected singleton genes (not shown). (**b**–**d**) Individual MCODE clusters, numbered by Cytoscape Results Panel ranking: (**b**) Cluster 1, interferon-stimulated gene (ISG) network (*n* = 14); (**c**) Cluster 2, MHC class I antigen-presentation cluster (*n* = 5); (**d**) Cluster 3, ribosomal cluster (*n* = 4).

Of the 160 meta-DEGs submitted to STRING, 154 mapped to STRING identifiers (96.2%); the six unmapped genes (*Mx2*, *AU020206*, *Mx1*, *Clec7a*, *Iigp1c*, *Rpl14-ps1*) lacked STRING annotations and were excluded from network construction. The 154-gene PPI network (194 edges) resolved into 83 connected components. Of these, 77 genes had no interaction partner at this confidence threshold and therefore formed single-gene components. The remaining 77 genes were grouped into six components: one large component containing 66 genes, one three-gene component (*Plin2*, *Xdh*, *Cybb*), and four two-gene components (*Myo18b*-*Myo1f*, *Ggta1*-*Gla*, *Sall1*-*Chd4*, *Cd72*-*Cd22*) (Figure 3a). Among the 14 curated BTK/NOX2 panel genes, only *Cybb* was present among the meta-DEGs submitted to STRING; it formed the three-gene component noted above but was not identified within any MCODE-identified cluster.

MCODE clustering, applied to the full network, identified three candidate clusters (23 genes total; Section 2.4). All three were retained for functional annotation given the small number identified (Section 2.5). Functional enrichment was performed independently for each of the three largest clusters against GO Biological Process, KEGG, and Reactome, using the 16,636-gene universe (full results in Table S10).

**Figure 4:**
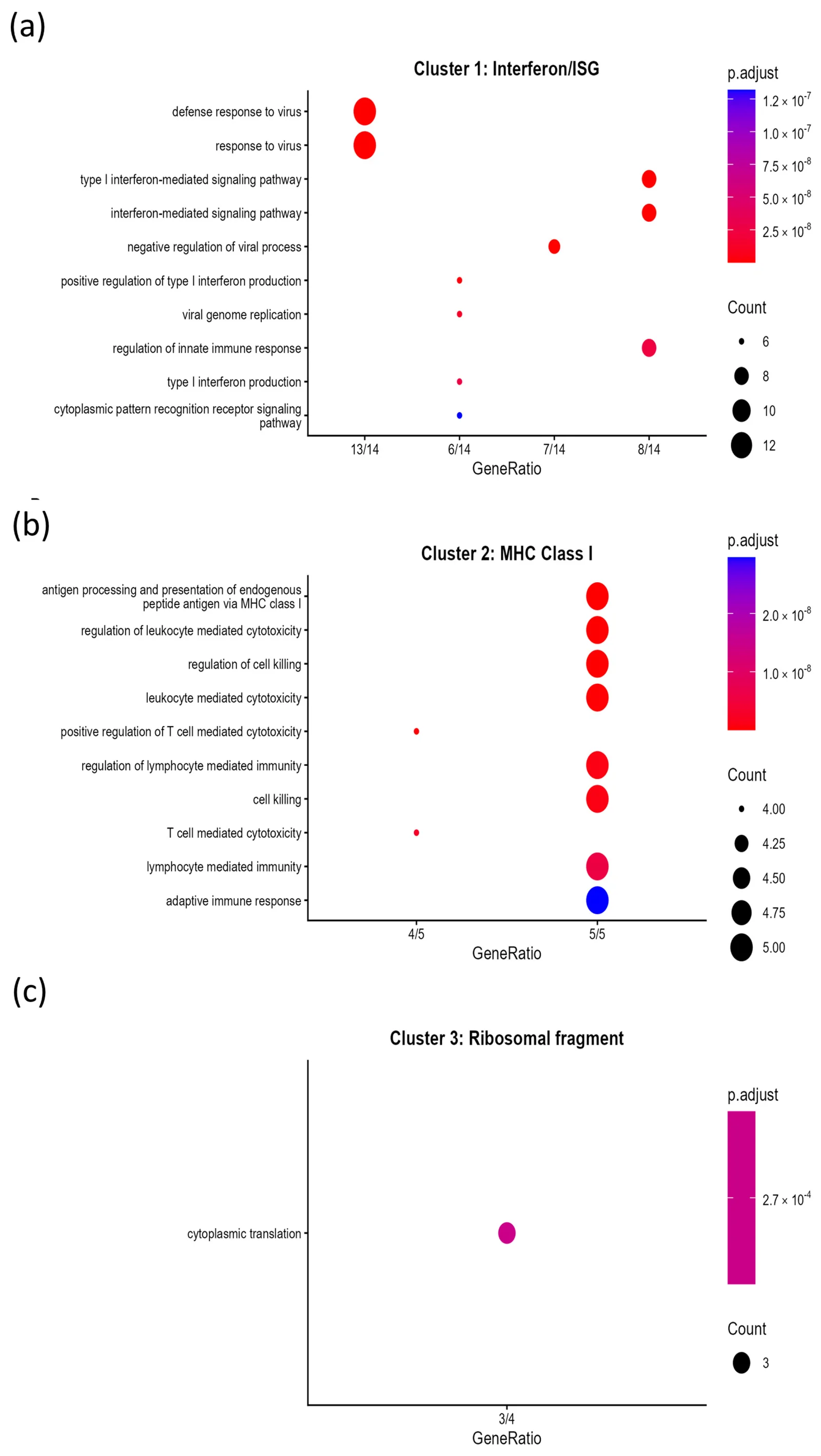
GO Biological Process enrichment dot plots for the three MCODE clusters with cluster-level enrichment performed: (**a**) Cluster 1, ISG network (*n* = 14); (**b**) Cluster 2, MHC class I cluster (*n* = 5); (**c**) Cluster 3, ribosomal cluster (*n* = 4). Terms were simplified using clusterProfiler’s simplify () function (similarity cutoff = 0.7) prior to plotting; dot size: gene count; dot color: BH-adjusted *p*-value. Complete unsimplified GO Biological Process, KEGG, and Reactome results for each cluster are provided in Table S10.

Cluster 1 (*n* = 14; Figure 3b) comprised interferon-stimulated genes (*Eif2ak2*, *Oas1g*, *Oas2*, *Oas3*, *Usp18*, *Dhx58*, *Ifit2*, *Irf7*, *Oasl2*, *Stat1*, *Stat2*, *Rtp4*, *Rsad2*, *Cmpk2*). GO Biological Process enrichment identified defense response to virus as the top term (13/14 cluster genes; *p*adj= 1.15 × 10^−19^), followed by response to virus (13/14; *p*adj = 9.46 × 10^−19^) and type I interferon-mediated signaling pathway (8/14; *p*adj = 1.41 × 10^−14^) (Figure 4a). KEGG enrichment gave the same result: after excluding viral and immune-disease pathway annotations that reflect shared ISG membership rather than viral pathology (Section 2.5), the top surviving term was NOD-like receptor signaling pathway (6/11, *p*adj = 3.85 × 10^−7^) (Table S10). Reactome enrichment, based on the 3 of 14 cluster genes with mouse Reactome pathway annotations (*Eif2ak2*, *Irf7*, *Usp18*), identified ISG15 antiviral mechanism as the top term (2/3, *p*adj = 1.41 × 10^−3^) (Table S10). Cluster composition was identical at node score cutoffs 0.2 and 0.3; at the more permissive cutoff of 0.1, the cluster fragmented into two smaller subclusters (eight and six genes) that together recovered all 14 original genes (Table S8). This indicated that the ISG cluster was a robust feature of the network at a finer level of internal structure.

Cluster 2 (*n* = 5; Figure 3c) comprised MHC class I genes (*H2-D1*, *H2-Q6*, *H2-K1*, *H2-Q4*, *Tap1*). All 5 cluster genes contributed to the top GO term, antigen processing and presentation of endogenous peptide antigen via MHC class I (5/5; *p*adj = 3.45 × 10^−13^), followed by regulation of leukocyte-mediated cytotoxicity (5/5; *p*adj = 7.25 × 10^−11^) (Figure 4b). KEGG enrichment supported this interpretation (Antigen processing and presentation, 5/5, *p*adj = 2.01 × 10^−9^). Reactome analysis yielded a consistent result (Antigen Presentation: Folding, assembly and peptide loading of class I MHC, 4/4, *p*adj = 2.00 × 10^−9^) (Table S10). Cluster composition was identical across all three node score cutoffs tested (0.1, 0.2, and 0.3), indicating this cluster is the most robust of the three clusters.

Cluster 3 (*n* = 4; Figure 3d) comprised ribosomal protein genes (*Rps7*, *Rps18*, *Rpl19*) and *Ptma*. GO Biological Process enrichment returned a single significant term, cytoplasmic translation (3/4, *p*adj = 2.73 × 10^−4^) (Figure 4c), reflecting the cluster’s small size. KEGG enrichment identified ribosome (3/3, *p*adj = 3.97 × 10^−5^; Coronavirus disease excluded as a viral and immune-disease annotation artifact), and Reactome identified SRP-dependent cotranslational protein targeting to membrane as the top term (3/3, *p*adj = 7.81 × 10^−6^) (Table S10). Unlike Clusters 1 and 2, this cluster was not identified at the more permissive node score cutoff of 0.1, making it the least robust of the three clusters. Its enrichment result should accordingly be interpreted as suggestive rather than definitive (Table S8).

### 3.3. Global Functional Enrichment of Meta-DEGs Reveals Dominant Interferon and Translational Programs

**Figure 5:**
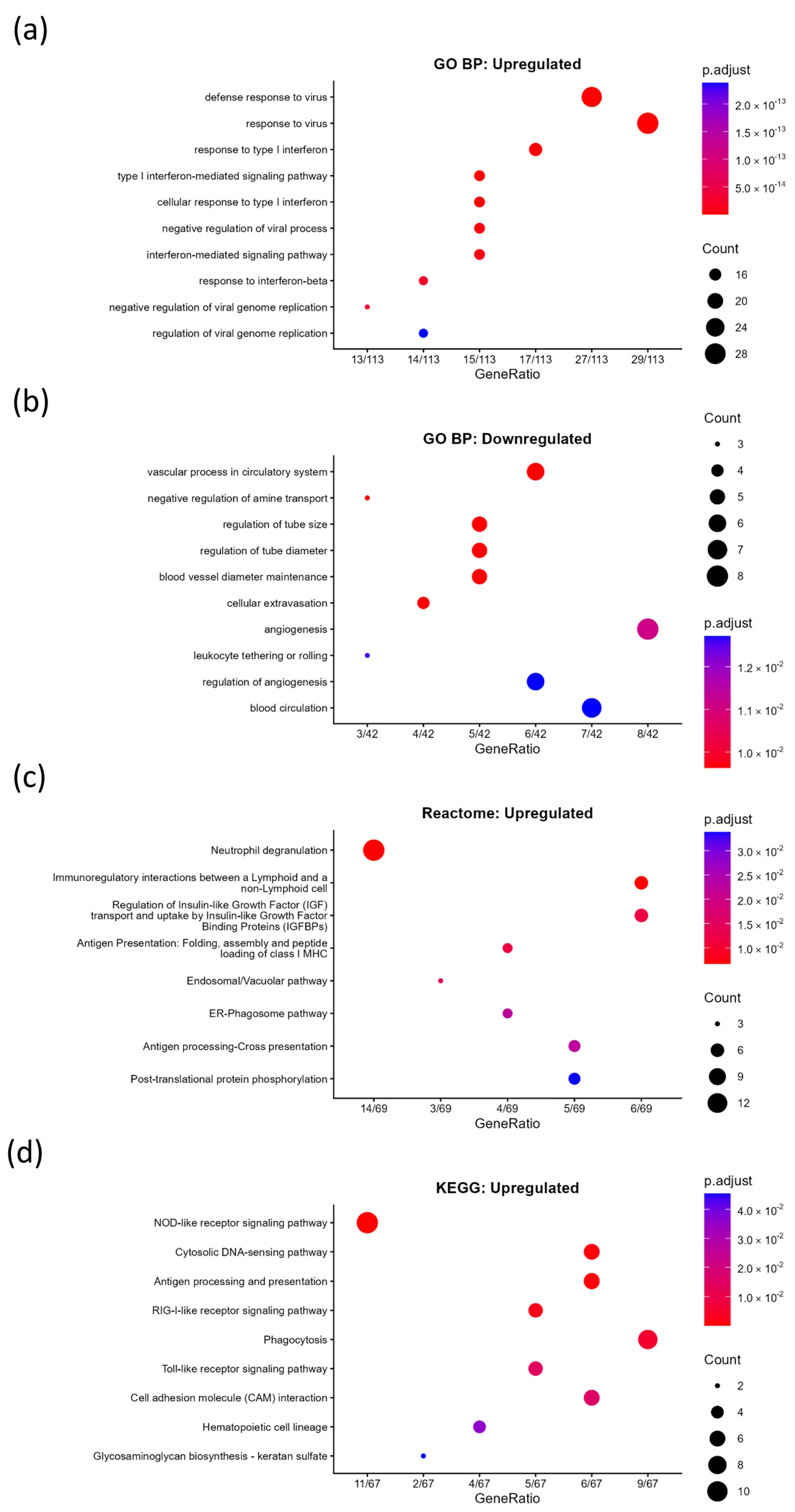
Functional enrichment analysis of meta-differentially expressed genes at 7 dpi. (**a**) GO Biological Process enrichment of upregulated meta-DEGs (*n* = 117). (**b**) GO Biological Process enrichment of downregulated meta-DEGs (*n* = 43). (**c**) Reactome enrichment of upregulated meta-DEGs. (**d**) KEGG enrichment of upregulated meta-DEGs (viral and immune-disease annotation artifacts excluded; see Methods 2.5 and Discussion, interferon signaling). Dot size: gene count; dot color: BH-adjusted *p*-value. Full GO Biological Process, KEGG, and Reactome enrichment results for both gene sets are provided in Table S11.

ORA of the 117 upregulated and 43 downregulated meta-DEGs was performed using GO Biological Process, KEGG, and Reactome databases (Figure 5).

**Upregulated meta-DEGs.** GO Biological Process (BP) enrichment of the 117 upregulated meta-DEGs (113 genes with GO BP annotation) yielded 230 significant terms after BH correction (*p*adj < 0.05), dominated by innate immune and interferon-related processes. The top-ranked terms were response to virus (29/113, *p*adj = 1.26 × 10^−19^) and defense response to virus (27/113, *p*adj = 1.26 × 10^−19^), followed by response to type I interferon (17/113, *p*adj = 1.16 × 10^−18^) and type I interferon-mediated signaling pathway (15/113, *p*adj = 2.61 × 10^−16^) (Figure 5a). These terms reflect the co-annotation of post-TBI ISGs under antiviral GO categories in the database; they do not indicate viral infection in any of the four experimental datasets, which used sterile injury models throughout. Reactome enrichment analysis identified eight significant terms, with neutrophil degranulation showing the strongest enrichment (14/69, *p*adj = 0.0067), along with pathways related to antigen presentation and phagocytosis (Figure 5c). After removal of viral and immune-disease pathway annotations, KEGG enrichment identified nine significant pathways. The NOD-like receptor signaling pathway was the top non-artifactual pathway (11/67, *p*adj = 7.76 × 10^−6^), followed by phagocytosis (9/67 genes), antigen processing and presentation, cytosolic DNA-sensing pathway, and the RIG-I-like receptor signaling pathway (Figure 5d).

**Downregulated meta-DEGs.** GO BP enrichment of the 43 downregulated meta-DEGs (42 genes with GO BP annotation) yielded 21 significant terms. The largest term was Angiogenesis (8/42). A vascular-regulation cluster came next: vascular process in circulatory system, regulation of tube size/diameter, blood vessel diameter maintenance, cellular extravasation, and negative regulation of amine transport (all *p*adj = 0.0096 to 0.0099) (Figure 5b). No KEGG pathway reached statistical significance among downregulated meta-DEGs (lowest *p*adj = 0.234), nor did any Reactome pathway (lowest *p*adj = 0.183). Full GO Biological Process, KEGG, and Reactome enrichment results for both gene sets are provided in Table S11.

**Cross-database convergence.** Across GO BP and KEGG, but not Reactome, the leading biological program in the upregulated meta-DEG set was innate immune and interferon signaling, consistent with the ISG cluster identified at the network level (MCODE Cluster 1, Section 3.2).

### 3.4. Exploratory Co-Expression Network Analysis

WGCNA of GSE283560 (*n* = 11) identified 12 co-expression modules among 13,388 genes, and none was associated with TBI status (strongest module r = 0.60, family-wise permutation *p* = 0.27). Sample clustering did not follow injury status, whereas the two largest modules were strongly correlated with a vascular/fibroblast marker score (r = 0.69 and −0.70), suggesting that network structure was influenced by differences in the sample composition (Figure S7, Table S12). The 14 BTK/NOX2 panel genes were spread over five modules and did not form a shared module (Table S13).

Removing GSM8666331, the lowest-depth sample, produced one module (greenyellow, 210 genes) that correlated with TBI status (r = 0.92, family-wise *p* = 0.008). The module was enriched for response to interferon-beta (BH-adjusted *p* = 1.1 × 10^−8^), positive regulation of defense response (*p* = 1.8 × 10^−8^), and cytoplasmic translation (*p* = 1.2 × 10^−8^), and contained *Cybb* (Figure S8, Tables S14 and S15). A significant module also appeared after removing GSM8666365 (r = 0.96, family-wise *p* = 0.008), but in none of the other nine leave-one-out runs (Table S15). Because significance depended on removing one of the two lowest-depth TBI samples, this module is exploratory, and no BTK/NOX2 conclusion was drawn from WGCNA.

### 3.5. Gene Set Enrichment Analysis Highlights Interferon, Translation and a Limited Lipid Signal

**Figure 6:**
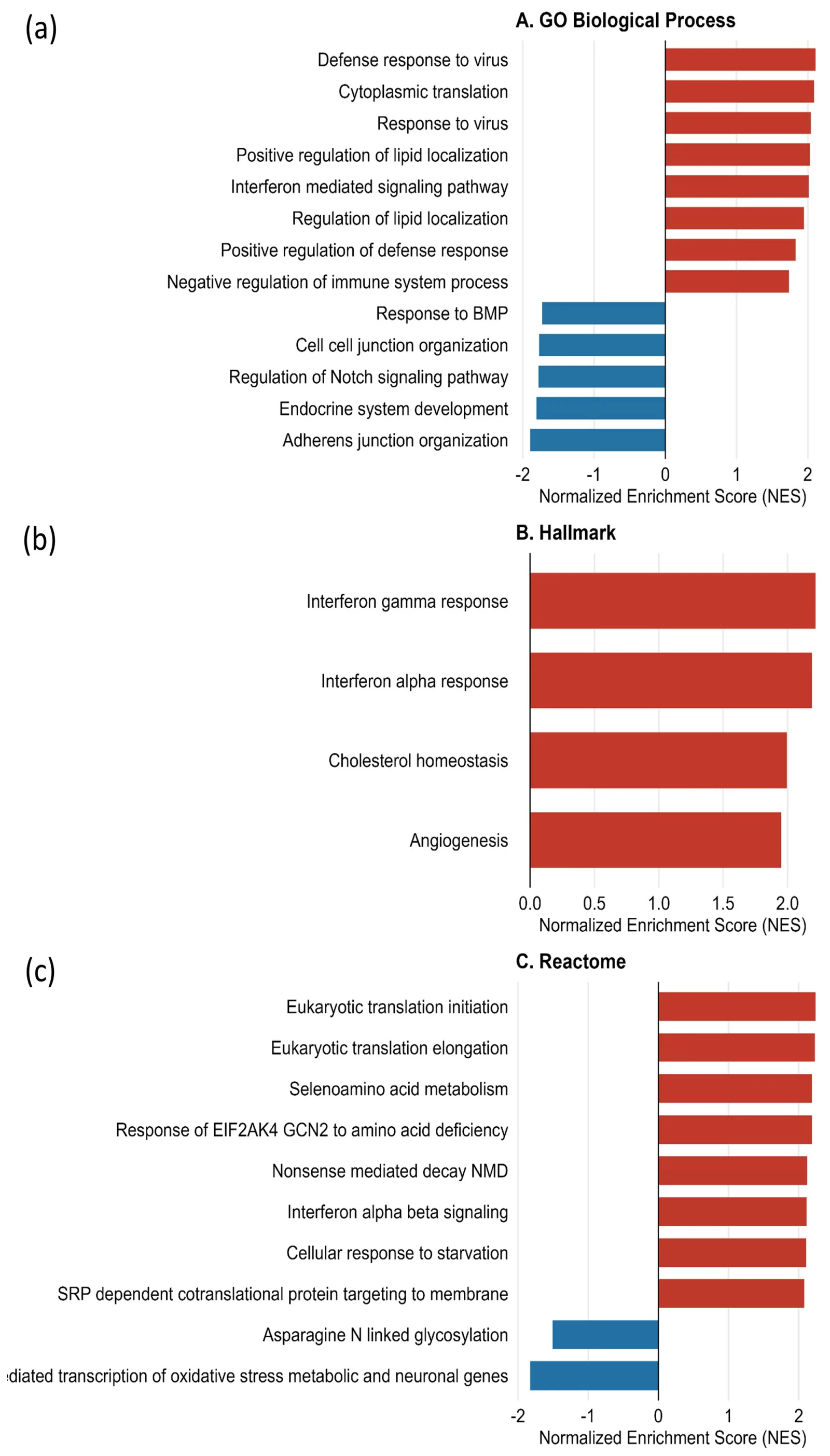
Gene set enrichment analysis at 7 dpi. (**a**) Top significant GO Biological Process terms. Terms with “viral” in their names were omitted because they overlap with defense responses to viruses. (**b**) All four significant Hallmark gene sets. (**c**) Top eight positive and both negative significant Reactome pathways. Three virus-named terms driven by ribosomal proteins are not shown (see text). Most translation-related terms share the same ribosomal genes. Red bars: positive NES; blue bars: negative NES.

GSEA was performed on the meta-ranked list of 16,636 genes. All genes mapped to ENTREZ IDs and were ranked. Three collections were tested: GO Biological Process (3748 terms tested, 54 significant at adjusted *p* < 0.05), Hallmark (50 tested, 4 significant), and Reactome (1032 tested, 26 significant) (Figure 6a–c). A positive NES indicates that the genes within a given set are preferentially ranked toward the TBI-associated end of the gene list. Of the 54 significant GO terms, 37 had positive NES and 17 had negative.

**GO Biological Process.** The 37 positive NES terms were allocated into four groups. Twelve terms were related to interferon and viral response, led by defense response to virus (NES = 2.11, *p*adj = 1.3 × 10^−6^) and response to virus (NES = 2.04, *p*adj = 3.7 × 10^−6^). Fifteen terms were related to cytokine production and innate immune regulation (NES = 1.64 to 1.91). Seven terms were related to lipid and isoprenoid processes, such as positive regulation of lipid localization (NES = 2.03, *p*adj = 2.7 × 10^−3^). Cytoplasmic translation was also significantly enriched (NES = 2.09, *p*adj = 1.7 × 10^−4^) and ranked as the fourth strongest GO term overall. Two other terms were protein containing complex remodeling and response to nutrients. The virus-related terms were driven by interferon-stimulated genes, rather than viral infection, because all four datasets used sterile injury (Section 3.3) (Figure 6a).

Seventeen terms demonstrated negative enrichment (NES = −1.64 to −1.90). Five of these were related to cell–cell junctions and adhesion, including adherens junction organization (NES = −1.90, *p*adj = 0.017). Four covered Notch, BMP, and SMAD signaling. Two covered muscle cell differentiation. Six other terms were not grouped, including glomerulus development, megakaryocyte development, and endoplasmic reticulum to Golgi vesicle transport.

**Hallmark.** All four significant sets showed positive enrichment. Interferon Gamma Response (NES = 2.22, *p*adj = 4.2 × 10^−10^) and Interferon Alpha Response (NES = 2.19, *p*adj = 5.0 × 10^−7^) were the strongest. Cholesterol Homeostasis (NES = 2.00, *p*adj = 0.0050) and Angiogenesis (NES = 1.95, *p*adj = 0.0053) were also significant. These two gene sets were supported by a relatively small number of contributing genes. The leading edge, the genes that drove the score, had 10 of 72 genes in Cholesterol Homeostasis (*Lpl*, *Fabp5*, *Anxa5*, *Lgals3*, *Gusb*, *Cxcl16*, *Cd9*, *Scd2*, *Plaur*, *Atf3*) and 5 of 31 genes in Angiogenesis (*Lpl*, *Spp1*, *Nrp1*, *App*, *Lrpap1*). *Lpl* and *Cd9* are described in disease-associated microglia [10]. *Spp1* is also raised in a subset of microglia after TBI [12]. These findings are therefore more consistent with a lipid-handling signal than with evidence of Angiogenesis. The other 46 Hallmark sets were not significant (Figure 6b). Full results are presented in Table S16.

**Reactome.** Twenty-four pathways were positive, and two were negative (Figure 6c). Eukaryotic Translation Initiation (NES = 2.24, *p*adj = 6.5 × 10^−8^) and Elongation (NES = 2.23, *p*adj = 2.4 × 10^−7^) were the top pathways. Selenoamino Acid Metabolism, Response of EIF2AK4 (GCN2) to Amino Acid Deficiency, and Nonsense-Mediated Decay followed (NES = 2.12 to 2.19). Most of these terms share the same ribosomal protein genes and therefore are not independent results. The following interferon pathways were also significant: Interferon Alpha/Beta Signaling (NES = 2.11, *p*adj = 1.1 × 10^−3^), Interferon Signaling (NES = 1.99, *p*adj = 1.3 × 10^−3^), and Interferon Gamma Signaling (NES = 1.99, *p*adj = 0.019). Plasma Lipoprotein Remodeling was significant as well (NES = 1.98, *p*adj = 0.011), which fits the lipid signal in the Hallmark result.

Three significant Reactome terms carry virus names: Influenza Infection (NES = 2.08, *p*adj = 0.0012), SARS-CoV-1 Modulates Host Translation Machinery (NES = 2.06, *p*adj = 0.0027), and SARS-CoV-2 Modulates Host Translation Machinery (NES = 2.02, *p*adj = 0.030). The majority of their leading-edge genes were ribosomal proteins (81 of 88, 33 of 35, and 33 of 34 genes). These terms reflect the ribosomal signal and do not indicate viral infection or an interferon effect. They were excluded from Figure 6.

Two pathways were negative: Asparagine N-linked Glycosylation (NES = −1.51, *p*adj = 0.012) and FOXO-mediated Transcription of Oxidative Stress, Metabolic and Neuronal Genes (NES = −1.83, *p*adj = 0.033). The full results are presented in Table S16.

### 3.6. The BTK/NOX2 Panel Shows a Single Significant Gene and No Coordinated Panel Signal

**Figure 7:**
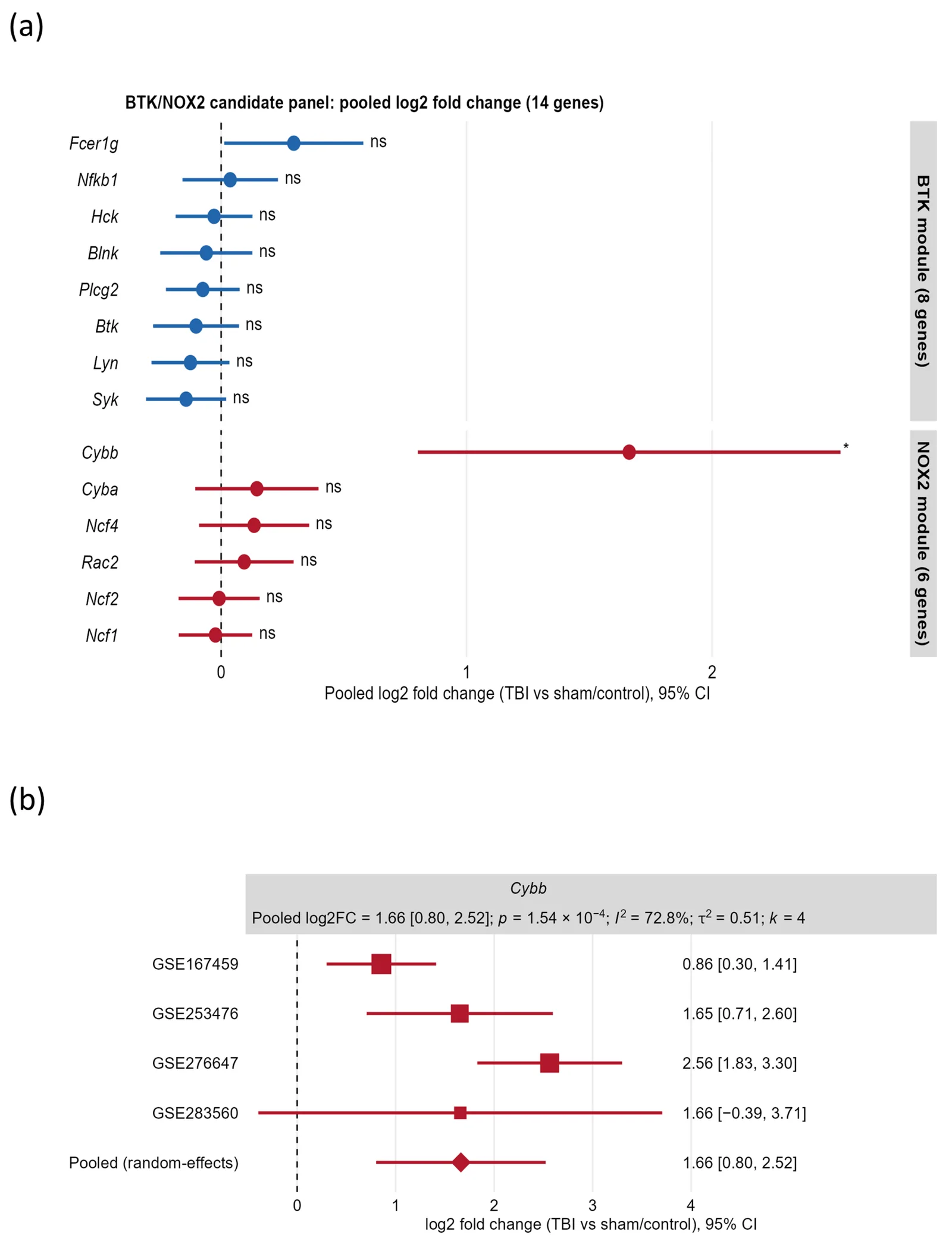
BTK/NOX2 panel analysis at 7 dpi. (**a**) Pooled log_2_ fold change (TBI versus sham or control) with 95% CI for the 14 panel genes, grouped by gene group (BTK, *n* = 8; NOX2, *n* = 6). Asterisks denote the BH-adjusted *p*-value: * *p* < 0.05; ns, not significant. Only *Cybb* reaches significance. (**b**) Forest plot for *Cybb* in the four datasets. The square area is proportional to the random-effects weight, and the diamond is the pooled estimate. The panel title gives the pooled log_2_FC [95% CI], the unadjusted *p*-value, *I*^2^, τ^2^, and the number of datasets (*k*). The BH-adjusted *p*-value (0.0188) is given in Table S17.

Each of the 14 panel genes was first evaluated within the genome-wide meta-analysis (Figure 7a; Table S17). Only *Cybb* remained significant after Benjamini–Hochberg (BH) correction across all 16,636 analyzable genes (pooled log_2_FC = 1.66, 95% CI 0.80 to 2.52; *p* = 1.54 × 10^−4^; BH-adjusted *p* = 0.0188; *I*^2^ = 72.8%; *k* = 4). All of the remaining 13 genes had a BH-adjusted *p* of 0.9998. *Fcer1g* reached a nominal *p* of 0.041 (pooled log_2_FC = 0.30, 95% CI 0.01 to 0.58; *I*^2^ = 43.6%), which did not survive correction, and *Btk* was unchanged (pooled log_2_FC = −0.10).

Coordinated regulation was assessed with three aggregate tests (Table 2). A gene set test of the 14-gene panel against the meta-ranked list was not significant (NES = 1.04, *p* = 0.417). One-sample *t*-tests of pooled log_2_FC against zero were likewise not significant for the BTK gene group (*n* = 8; mean log_2_FC = −0.025; t(7) = −0.50, *p* = 0.631) or the NOX2 gene group (*n* = 6; mean log_2_FC = 0.334; t(5) = 1.25, *p* = 0.266). The positive NOX2 mean was driven largely by *Cybb*, and the remaining five NOX2 genes had a mean log_2_FC of approximately 0.07. Because these *t*-tests weight all genes equally and have few degrees of freedom, they have limited power, and a non-significant result indicates that no group shift was detected rather than that the gene groups are unrelated.

*Cybb* was elevated in TBI relative to sham or control in all four datasets (Figure 7b). Dataset-level estimates ranged from 0.86 (GSE167459) to 2.56 (GSE276647), with random-effects weights of 32.5% (GSE167459), 25.9% (GSE253476), 29.5% (GSE276647), and 12.0% (GSE283560). The 95% CIs excluded zero in three datasets. The CI for GSE283560 crossed zero (log_2_FC = 1.66, 95% CI −0.39 to 3.71), indicating the greatest uncertainty among the datasets. The dispersion of estimates indicated between-study heterogeneity (*I*^2^ = 72.8%; τ^2^ = 0.51) without any change in direction. Because only four studies were included, the estimate of between-study heterogeneity is itself imprecise.

*Fcer1g* did not reach significance in the primary analysis; its dataset-level estimates were 0.24, 0.53, 0.06 and −0.07 (Figure S6, upper panel). Pooling unshrunk estimates (Section 2.3.1) produced a larger and significant pooled effect (log_2_FC = 0.51, 95% CI 0.31 to 0.72; BH-adjusted *p* = 5.9 × 10^−5^; *I*^2^ = 0%; Figure S6, lower panel). Under this approach, GSE253476 contributed 71.5% of the total weight, compared with 41.5% in the primary analysis. The Fcer1g result was therefore sensitive to the choice of effect-size estimator and should not be interpreted as a finding of the primary analysis.

*Cybb* remained significant when unshrunk estimates were pooled (BH-adjusted *p* = 0.00033; Section 2.3.1) and when the analysis was restricted to the 11,223 genes represented in all four datasets (BH-adjusted *p* = 0.0143; Section 2.3.2; Table S5). In the leave-one-dataset-out analysis (Section 2.3.3; Table S6), the pooled *Cybb* estimate remained positive in all four re-pooled analyses (log_2_FC = 1.19 to 2.11; nominal *p* = 3.0 × 10^−8^ to 0.0059). After BH correction across all genes tested in each re-pooled analysis, *Cybb* was significant when GSE167459 (BH-adjusted *p* = 7.8 × 10^−6^) or GSE276647 (BH-adjusted *p* = 0.025) was excluded, but not when GSE253476 (BH-adjusted *p* = 0.700) or GSE283560 (BH-adjusted *p* = 0.086) was excluded. Each re-pooled analysis included only three datasets, resulting in reduced statistical power. Across the four analyses, the global meta-DEG count ranged from 77 to 311, compared with 160 in the primary analysis. In the leave-one-sample-out analysis (Section 2.3.4; Table S7), *Cybb* remained significant in three of the four exclusion analyses (BH-adjusted *p* = 0.0087 to 0.017); however, it was not significant after removal of the flagged sample from GSE167459 (BH-adjusted *p* = 0.075). By contrast, Fcer1g was significant only without TBI_Iso_1 (*p*adj = 0.025) in GSE276647. The direction of the *Cybb* effect was therefore stable across analyses, whereas statistical significance after multiple-testing correction varied depending on which datasets were included.

## 4. Discussion

This meta-analysis of four independent mouse microglial RNA-seq datasets demonstrated that microglia at 7 days after injury share a reproducible transcriptional program, despite differences in injury model, brain region, and isolation strategy. The program combines interferon signaling, antigen presentation, and lipid handling, together with a partial reduction in homeostatic genes. We regard this genome-wide response as the principal finding of the study. The BTK/NOX2 panel was examined as a secondary question, and it yielded a single significant gene, *Cybb*, without any coordinated signal across the panel as a whole.

Interferon signaling was the most consistent result. Gene set enrichment, over-representation analysis, and the largest network cluster all detected it, but they share the same meta-analysis result, so they are convergent views, not independent validation. A type I interferon response in microglia after TBI has been reported in individual studies [11], and this analysis shows that it is reproducible across datasets. The viral terms among the enriched categories reflect the annotation of interferon-stimulated genes and do not indicate infection, because all datasets used sterile injury. Sterile tissue injury releases nucleic acids and other damage-associated molecules from damaged cells. These signals can activate the cyclic GMP-AMP synthase (cGAS)–stimulator of interferon genes (STING) pathway, leading to type I interferon production; activation of this pathway has also been demonstrated in the brain after TBI [59,60]. MHC class I genes were also upregulated alongside this transcriptional program, suggesting coordinated induction of antigen-processing machinery.

A lipid-handling signal was the second feature of the response. *Lpl* and *Itgax*, which are linked to DAMs [10], were among the most upregulated genes. *Spp1* [12] was also upregulated, and lipid-related gene sets were enriched. Because the Angiogenesis signal was driven by a small number of overlapping lipid-related and DAM-associated genes, we interpret this finding as reflecting lipid-handling processes rather than vascular growth. Together, these findings are consistent with a DAM-like state in post-TBI microglia. Such a state may reflect the role of microglia in clearing lipid-rich debris generated by damaged tissue. In the aging brain, microglia that accumulate lipid droplets are defective in phagocytosis, produce high levels of ROS, and secrete proinflammatory cytokines [61].

Homeostatic identity was reduced but not lost. *Cx3cr1* and *Sall1* were lower, whereas *P2ry12*, *P2ry13*, and *Gpr34* were not significant, which fits a partial departure from the resting state. The vascular and junction terms among the downregulated genes were primarily driven by a relatively small number of genes and were not interpreted biologically. Homeostatic microglial genes depend on TGF-β signaling [62]. Inactivation of Sall1 has been reported to convert resting microglia into inflammatory phagocytes [63].

Increased expression of translation-related genes was also prominent. However, many of the enriched terms were driven by overlapping ribosomal protein genes and therefore likely reflect a single underlying biological signal rather than multiple independent processes. Accordingly, this finding was interpreted more cautiously than the interferon-related enrichment. In addition, activation of immune cells is accompanied by greater ribosome biogenesis and protein synthesis, which supports cytokine production and cell growth [64,65].

Within the BTK/NOX2 panel, *Cybb* was the only gene that remained significant after correction for multiple testing, and the panel did not shift. The data therefore supports a gene-level change but no detectable pathway shift. *Fcer1g*, which encodes the ITAM-bearing adaptor of Fc receptors [55], was significant only with unshrunk estimates or one-sample exclusion and therefore is not interpreted as a finding. The absence of a change in *Btk* transcript was not unexpected, because BTK activity is regulated primarily through phosphorylation downstream of Fc receptors and Toll-like receptors rather than through changes in gene expression [15,56].

*Cybb*, which encodes gp91phox, the catalytic subunit of NOX2, was higher in TBI than in control animals in all four datasets. The magnitude of the increase varied across datasets, likely reflecting differences in injury model, brain region, and animal age. Accordingly, the pooled estimate should be interpreted primarily as evidence of a consistent direction of effect across studies rather than as a precise estimate of effect size. Network evidence did not support a NOX2-specific role for *Cybb*. The cross-dataset interaction network placed it in no cluster. Co-expression analysis of a single dataset (*n* = 11) was exploratory and did not inform conclusions concerning *Cybb*. Its results are presented in Section 3.4 and Supplementary Figures S7 and S8. NOX2 promotes proinflammatory microglial activation and neurodegeneration after experimental TBI [23]. In phagocytes, CYBB transcription is induced by interferon-gamma through Janus kinase 2 (JAK2) signaling [66], suggesting a potential mechanism linking interferon signaling to increased *Cybb* expression.

The literature does not clearly define the relationship between BTK and NOX2. BTK has been reported to restrain NOX2-dependent reactive oxygen species production in human neutrophils [24], whereas in antifungal defense it promotes neutrophil activation [25]. Consistent with these divergent findings, a recent review concluded that the relationship between BTK and NADPH oxidase remains unclear [26]. Transcript-level data can identify candidate genes within this pathway and provide a rationale for directly testing functional coupling between BTK and NOX2.

Several limitations should be acknowledged. First, the analysis included only four datasets, with three to eight animals per group, and was restricted to a single subacute time point. All datasets used male mice; therefore, the findings cannot be assumed to generalize to females, particularly because microglial responses to cortical injury may differ according to sex [67]. Second, the datasets differed in injury model, brain region, and microglial isolation strategy. Although such variation may reflect biologically meaningful differences in the microglial response to injury, including age-related effects [68], estimates of between-study heterogeneity based on only four studies are inherently imprecise [69].

Third, the network findings were sensitive to the MCODE node-score cutoff. At a cutoff of 0.1, the interferon-stimulated gene cluster separated into two subclusters, while the ribosomal cluster was no longer identified. These network results should therefore be viewed as one source of supporting evidence rather than as definitive findings. Fourth, the number of meta-DEGs also varied depending on the effect size estimator and the datasets included. In addition, GSE253476 contributed most of the weight to the *Fcer1g* estimate when unshrunk effects were pooled. *Cybb* lost significance after BH correction in the leave-one-dataset-out runs without GSE253476 or GSE283560. Four GSE167459 samples were not used in the primary analysis and formed a separate batch. When they were included, the *Cybb* result depended on whether the batch was modeled, and about one-third of the meta-DEG list changed (108 of 160 shared). When a batch term was included to account for the four additional samples, *Cybb* remained significant. *Cx3cr1* and *Sall1* kept their direction but lost significance, whereas interferon enrichment was stable. Accordingly, our interpretation emphasizes biological programs supported by multiple genes and complementary analytical approaches rather than isolated single-gene findings. Finally, a marker-based assessment did not indicate substantial peripheral immune-cell contamination, although one sample (GSM8666331, GSE283560, TBI group) deviated (lower *P2ry12*, higher *Cd3e*) and was retained in the primary analysis.

These findings identify a clear direction for further investigation. *Cybb* was consistently upregulated across all four datasets, whereas the *Btk* transcript abundance and the BTK/NOX2 panel as a whole showed no significant shift. This raises the question of whether BTK activity modulates NOX2 at the protein level. Use of a selective BTK inhibitor after TBI followed by measurements of phosphorylated BTK, NOX2 protein expression, and superoxide levels in isolated microglia in vitro would likely address this issue directly. Because interferon signaling was a major feature here, and interferon-gamma induces *CYBB* transcription in phagocytes [66], interferon-stimulated genes should be measured in the same cells, which would separate a BTK-dependent change in NOX2 from induction by interferon. In addition to direct BTK/NOX2 pathway modulation, complementary strategies that strengthen endogenous antioxidant defenses may also warrant investigation. For example, activation of the Nrf2/KEAP1 pathway has been shown to protect microglia from oxidative stress in other neurodegenerative settings and could represent an alternative or adjunct therapeutic approach [70]. The interferon and lipid-handling programs, including markers such as *Lpl*, also provide useful readouts for studies at earlier and later time points and in female animals. Such studies could determine the persistence of these transcriptional programs and whether they are conserved across sexes. Given that both NOX2 and BTK-related signaling have been implicated in neuroinflammation after experimental brain injury [16,23], direct investigation of their interaction is warranted.

## 5. Conclusions

This meta-analysis of four independent mouse microglial RNA-seq datasets collected 7 days after TBI identified a reproducible transcriptional response across two injury models, different brain regions, and different microglial isolation strategies. The predominant features were interferon signaling, antigen presentation, a lipid-handling profile consistent with disease-associated microglia, and a partial reduction in homeostatic gene expression. These findings, supported by differential expression, network, and enrichment analyses, represent the principal genome-wide response of the study.

Within the BTK/NOX2 panel, *Cybb* was the only gene that showed a significant increase, with a consistent direction of effect across all four datasets. In contrast, *Btk* and the panel as a whole showed no detectable transcriptional shift, supporting a gene-specific rather than pathway-level interpretation. Interferon signaling can induce CYBB in phagocytes and may therefore contribute to increased NOX2 expression after TBI, a hypothesis requiring direct testing. These findings identify *Cybb* as a candidate for mechanistic studies examining the relationship between BTK and NOX2 in post-injury microglia. Future studies should directly assess BTK phosphorylation, NOX2 activity, and interferon-stimulated genes, and determine whether these transcriptional patterns persist across other post-injury time points and in female animals.

## Figures and Tables

**Table 1:** Characteristics of the four bulk RNA-sequencing datasets included in the meta-analysis. CCI, controlled cortical impact; FPI, fluid percussion injury; FACS, fluorescence-activated cell sorting; dpi, days post-injury.

Dataset	Reference	Injury Model	Tissue/Region	Cell Isolation	*n* (Sham/Control)	*n* (TBI)	Timepoint
GSE276647	Izzy et al., 2025 [29]	CCI	Ipsilateral hemisphere	FACS-sorted microglia (4D4+ surface marker)	4	4	7d
GSE283560	Paladini et al., 2025 [30]	CCI	Whole brain	FACS-sorted microglia (CD11b+, CD45^mid/low^, tdTomato−)	3	8	7d
GSE253476	Dean et al., 2024 [31]	CCI	Perilesional cortex	FACS-sorted microglia (CD45lowCD11b+)	3	3	7d
GSE167459	Todd et al., 2021 [11]	FPI	Ipsilateral hemisphere	FACS-sorted microglia (CD11b+/CD45-intermediate)	3	3	7d

Sample sizes are those of the primary analysis. Flagged samples were excluded only in the leave-one-sample-out analysis (Section 2.3.4). GSE167459: ten microglia samples are listed in GEO; six were used in the primary analysis (Section 2.2.1).

**Table 2:** Aggregate-level tests of the 14-gene BTK/NOX2 signature at 7 dpi.

Test	Statistic	Mean Pooled log_2_FC	*p*-Value	Result
Custom-set GSEA (14 genes)	NES = 1.04	not applicable	0.417	Not significant
BTK gene group *t*-test (8 genes)	*t*(7) = −0.50	−0.025	0.631	Not significant
NOX2 gene group *t*-test (6 genes)	*t*(5) = 1.25	0.334	0.266	Not significant

The *t*-tests are unweighted and have limited power at this panel size (BTK gene group, df = 7; NOX2 gene group, df = 5).

## Data Availability

Data supporting the findings of this article are present in the main text and in the Supplementary Files. This study reanalyzed publicly available data from the Gene Expression Omnibus (GEO): GSE276647, GSE283560, GSE253476, GSE167459. All analysis scripts (01–21), the sample-selection tables for the four datasets, and a table of parameters are available at https://github.com/Yousif-Salah/tbi-microglia-7dpi-meta-analysis (accessed on 17 October 2026) (release v1.0.0; Zenodo DOI: https://doi.org/10.5281/zenodo.23212074).

## Supplementary Materials

The following supporting information can be downloaded at: https://www.mdpi.com/article/10.3390/biology15211915/s1, Table S1: Study screening records; Table S2: Detailed study/experimental parameters for the four included datasets; Table S3: Microglial purity marker expression across four datasets; Table S4: Full per-gene meta-analysis results across harmonized (apeglm) and unshrunk shrinkage conditions; Table S5: *k* = 4 sensitivity analysis results (11,223 genes, BTK/NOX2 panel included); Table S6: Leave-one-dataset-out results (genome-wide meta-DEG counts and *Cybb* statistics); Table S7: Leave-one-sample-out sensitivity analysis: global meta-DEG count, adjusted *p*-values of Cybb, Fcer1g and Btk, Hallmark interferon GSEA, and Cx3cr1/Sall1/Tjp1; Table S8: MCODE node score cutoff sensitivity analysis (0.1, 0.2, 0.3); Table S9: Complete meta-analysis results for all analyzed genes (random-effects model, *k* ≥ 2 datasets); Table S10: Complete GO Biological Process, KEGG, and Reactome enrichment results for each of the three MCODE network clusters; Table S11: Complete GO Biological Process, KEGG, and Reactome enrichment results for upregulated and downregulated meta-DEGs; Table S12: WGCNA module–trait statistics for the 11-sample and 10-sample analyses, and marker-gene expression per sample; Table S13: WGCNA module assignment and module membership (kME) of the 13,388 network genes, with the 14 BTK/NOX2 panel genes; Table S14: GO Biological Process enrichment of WGCNA modules for both analyses; Table S15: Leave-one-sample-out WGCNA results; Table S16: Complete GSEA results across GO Biological Process, Hallmark, and Reactome collections; Table S17: BTK/NOX2 panel gene-level meta-analysis results; Table S18: GSE167459 analysis with ten microglia samples. Figure S1: Sample-level quality control, GSE253476; Figure S2: Sample-level quality control, GSE283560; Figure S3: Sample-level quality control, GSE276647; Figure S4: Sample-level quality control, GSE167459; Figure S5: Microglial purity check across four datasets (*P2ry12*, *Cx3cr1*, *Ptprc*, *Cd3e*); Figure S6: Forest plots for *Fcer1g* in the primary (harmonized) and unshrunk analyses; Figure S7: WGCNA of GSE283560 with 11 samples; Figure S8: WGCNA after removal of GSM8666331; Figure S9: Principal component analysis of the ten GSE167459 microglia samples.

## Abbreviations

The following abbreviations are used in this manuscript:

BBB	Blood–brain barrier
BH	Benjamini–Hochberg (correction)
BP	Biological Process (GO)
BTK	Bruton’s tyrosine kinase
CCI	Controlled cortical impact
CD11b	Cluster of differentiation 11b
CD45	Cluster of differentiation 45
CI	Confidence interval
CNS	Central nervous system
DAM	Disease-associated microglia
dpi	Days post-injury
FACS	Fluorescence-activated cell sorting
FPI	Fluid percussion injury
GEO	Gene Expression Omnibus
GO	Gene Ontology
GSEA	Gene set enrichment analysis
I^2^	Heterogeneity index (percentage of variance due to between-study heterogeneity)
ISG	Interferon-stimulated gene
ITAM	Immunoreceptor tyrosine-based activation motif
KEGG	Kyoto Encyclopedia of Genes and Genomes
kME	Module eigengene-based connectivity
MCODE	Molecular Complex Detection
meta-DEG	Meta-analysis differentially expressed gene
MHC	Major histocompatibility complex
MLE	Maximum-likelihood estimate
NADPH	Nicotinamide adenine dinucleotide phosphate
NES	Normalized enrichment score
NOD	Nucleotide-binding oligomerization domain
NOX2	NADPH oxidase 2
ORA	Over-representation analysis
PCA	Principal component analysis
PPI	Protein–protein interaction
RNA-seq	RNA sequencing
REML	Restricted maximum likelihood
ROS	Reactive oxygen species
TBI	Traumatic brain injury
WGCNA	Weighted gene co-expression network analysis
