# Supplementary material for: Reproducible Interferon-Centered Microglial Response at 7 Days After Traumatic Brain Injury: A Transcriptomic Meta-Analysis with Focus on the BTK/NOX2 Panel
Source: Biology (Basel). 2026 Oct 22;15(21):1915. doi: 10.3390/biology15211915 (PMC13761848; doi:10.3390/biology15211915)
Supplement: Supplementary file 1 [file biology-15-01915-s001.zip › Supplementary Figures.pdf]

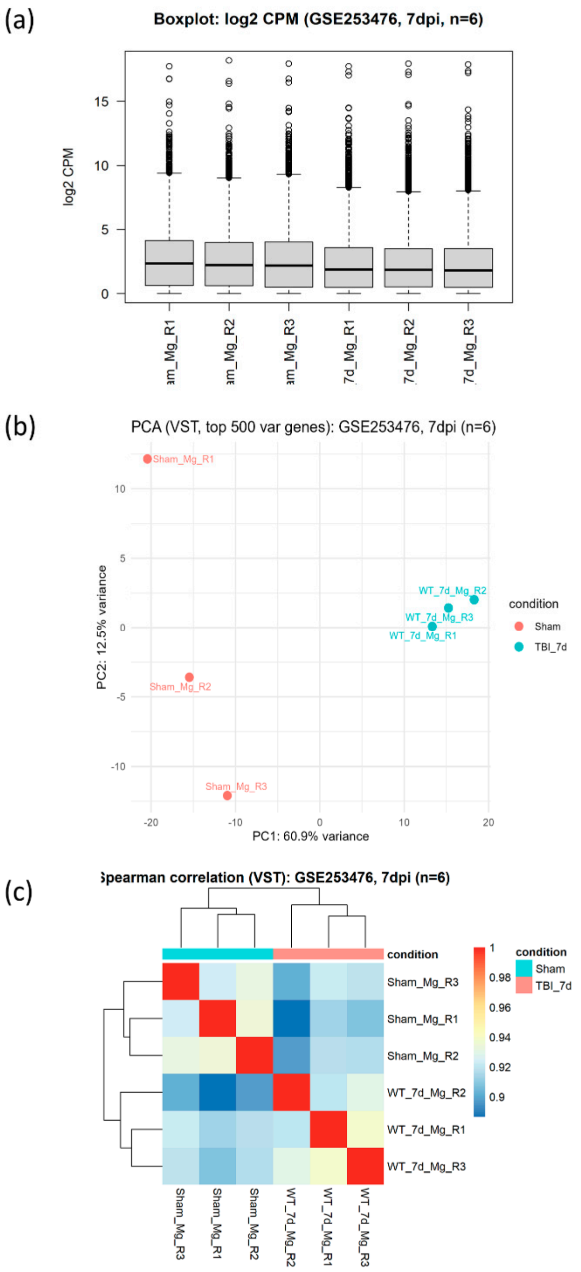

Figure S1. Sample-level quality control, GSE253476 (7 dpi, n = 6). (a) Boxplot of log2 CPM across samples; (b) PCA of variance-stabilized counts (top 500 variable genes), no sample separated clearly from its group; (c) Spearman correlation heatmap, within-group correlation 0.924–0.934. Sham_Mg_R3 (highest library size) was used for the leave-one-sample-out run (Section 2.2.3).

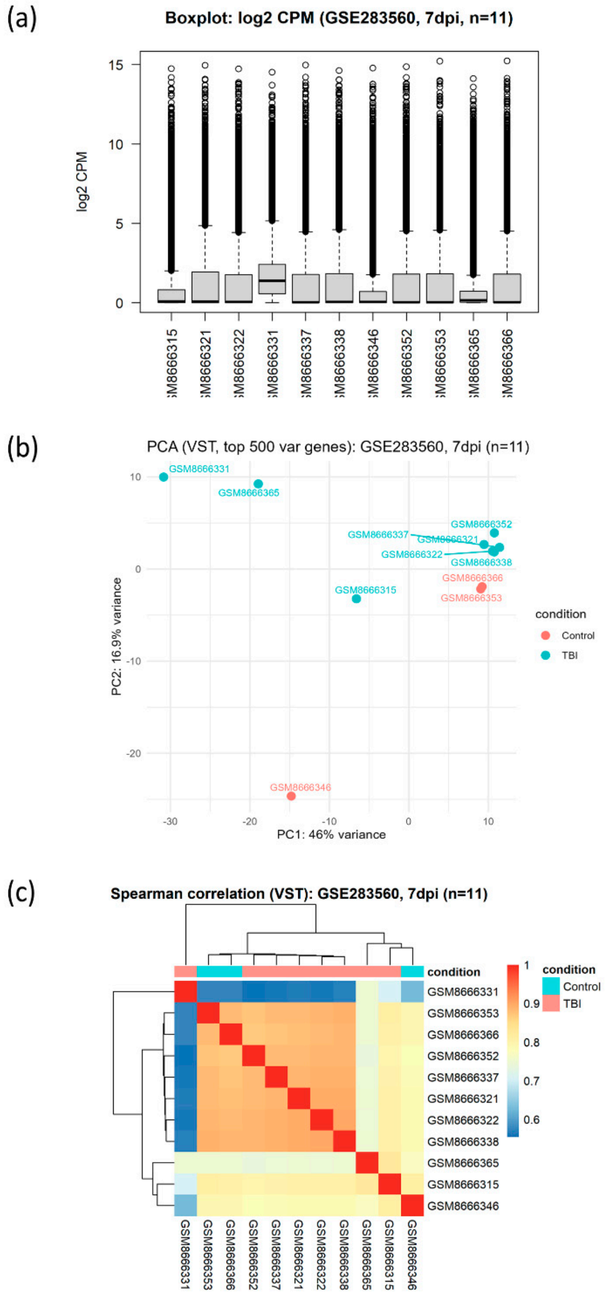

Figure S2. Sample-level quality control, GSE283560 (7 dpi, n = 11), counts from the three technical-replicate lanes summed. (a) Boxplot of log2 CPM; (b) PCA of variance-stabilized counts, showing GSM8666331 as the most distant sample (PC1 = −30.9); (c) Spearman correlation heatmap, showing GSM8666331 has collapsed correlation with the rest of the cohort, consistent with its markedly lower library size.

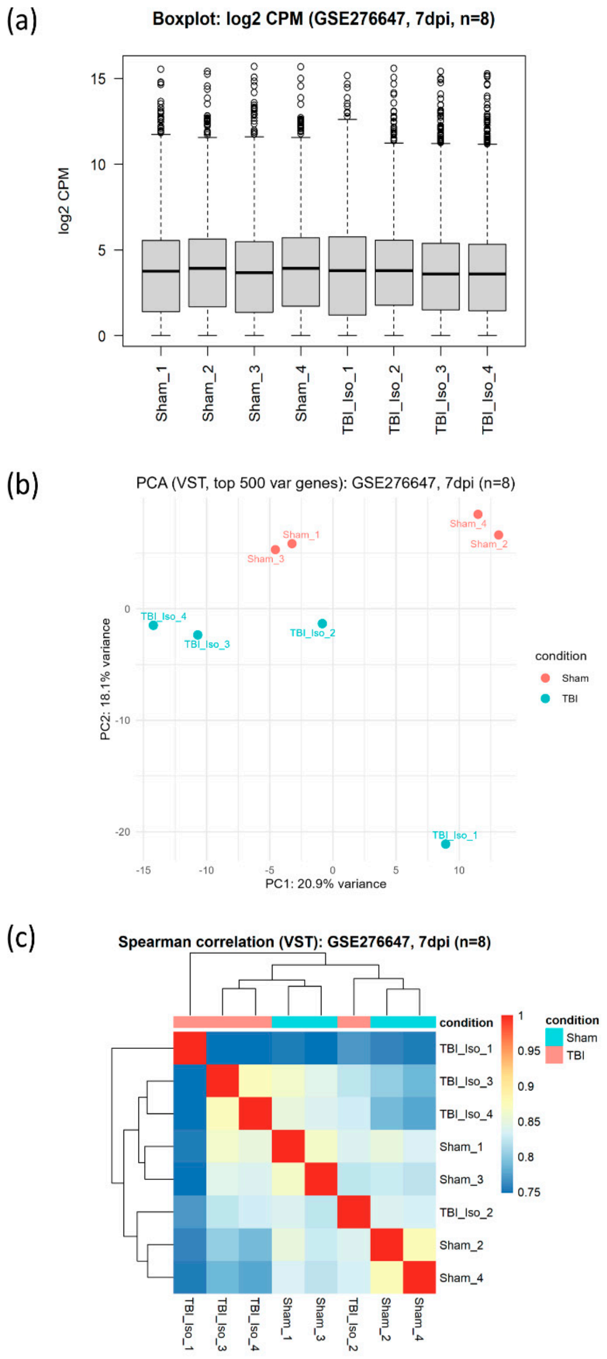

Figure S3. Sample-level quality control, GSE276647 (7 dpi, n = 8). (a) Boxplot of log2 CPM; (b) PCA of variance-stabilized counts, showing TBI_Iso_1 separating from other samples on PC2; (c) Spearman correlation heatmap, showing TBI_Iso_1 has reduced correlation with the rest of the cohort, independent of library size.

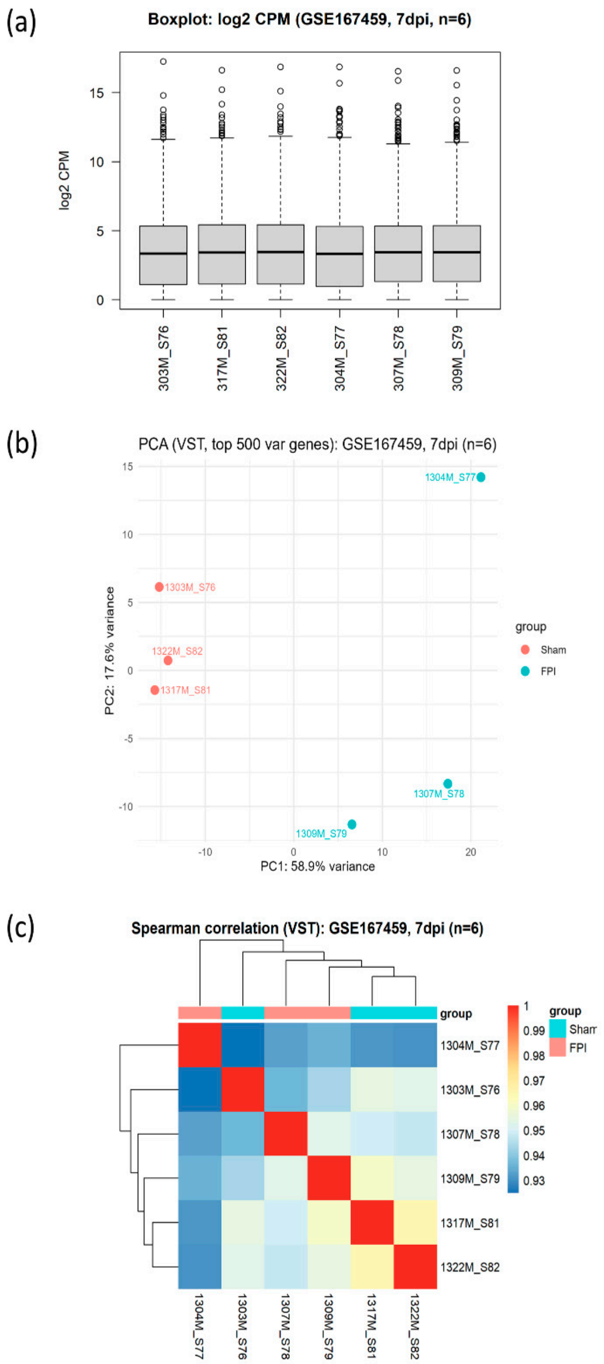

Figure S4. Sample-level quality control, GSE167459 (7 dpi, n = 6). (a) Boxplot of log2 CPM; (b) PCA of variance-stabilized counts, showing 1304M_S77 separating from the remaining five samples; (c) Spearman correlation heatmap, showing 1304M_S77 has reduced within-group correlation.

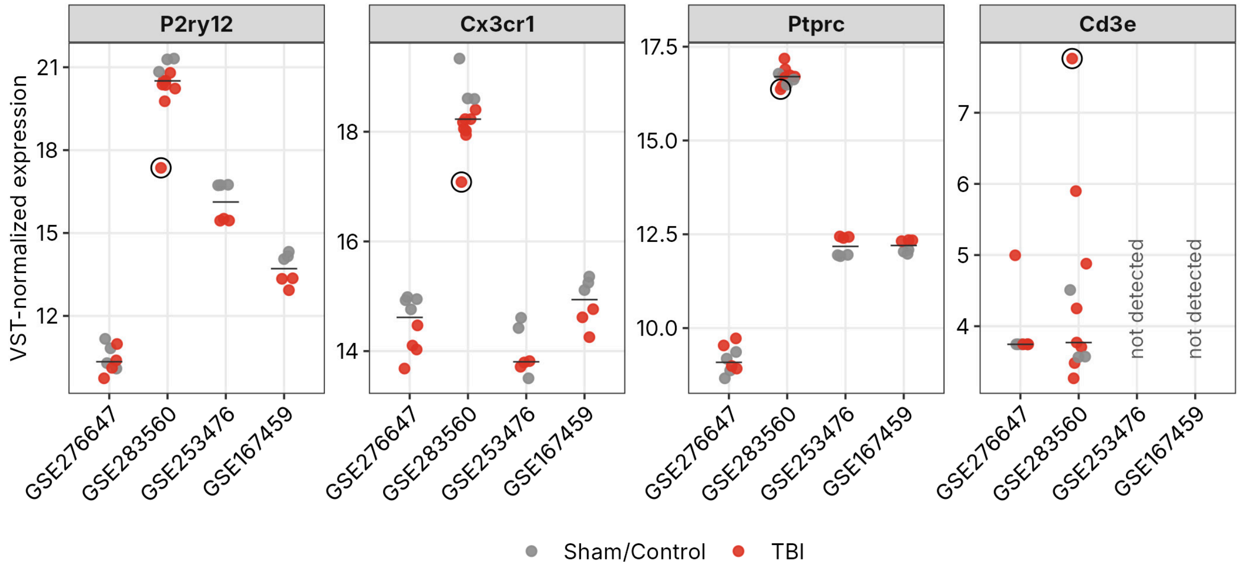

Figure S5. Microglial purity check across four datasets (P2ry12, Cx3cr1, Ptprc, Cd3e). Variance-stabilized expression of two microglial markers (P2ry12, Cx3cr1) and two peripheral immune-cell markers (Ptprc/Cd45, Cd3e) in each dataset's primary DESeq2 object (Section 2.2.2). Gray points, Sham/Control; red points, TBI; black ring, GSM8666331 (GSE283560, TBI); horizontal bars, group medians. Cd3e was not detected after low-count filtering in GSE253476 and GSE167459.

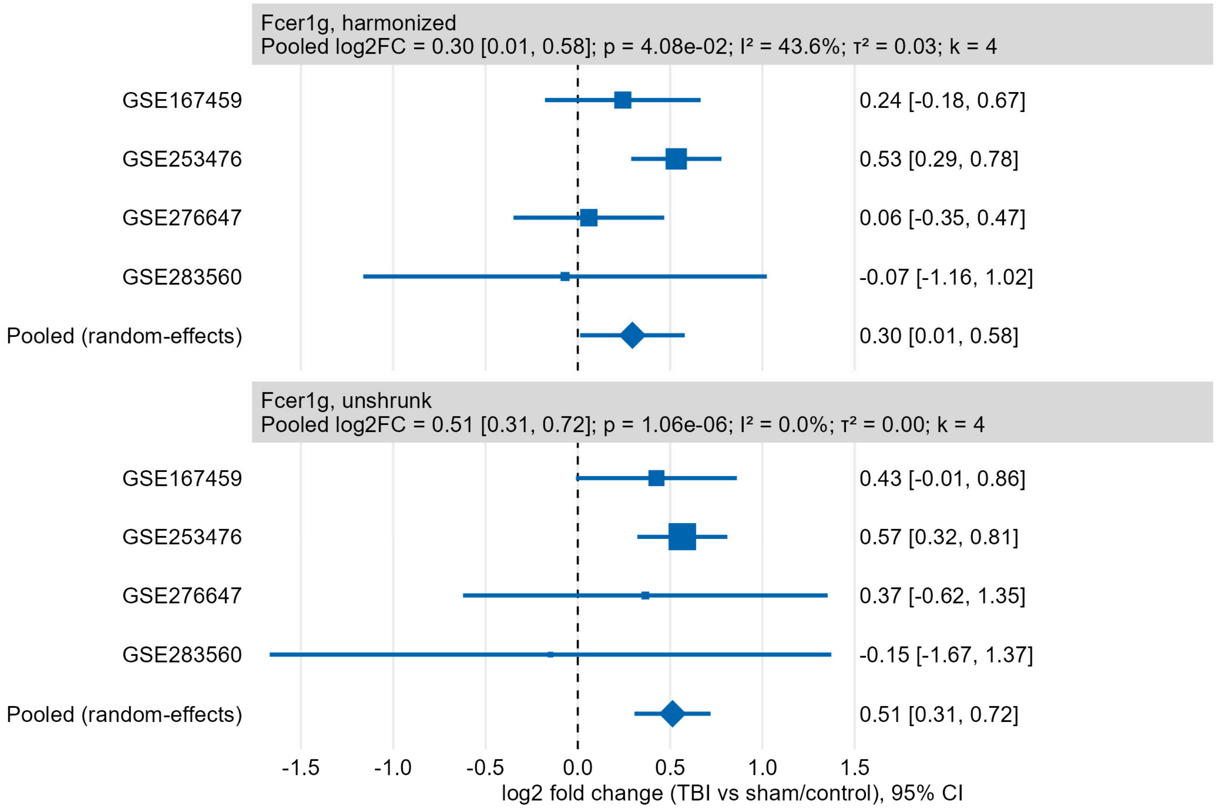

Figure S6. Forest plots for Fcer1g in the primary (harmonized) and unshrunk analyses. Upper panel, harmonized (BH-adjusted p = 0.9998); lower panel, unshrunk (BH-adjusted p = 5.9×10^{-5}). Points show dataset-level log2 fold change (TBI versus sham or control) with 95% CI. Square area is proportional to the random-effects weight, and diamonds show the pooled estimate. Panel titles give the pooled log2FC [95% CI], the unadjusted *p*-value, I^2, τ^2 and k.

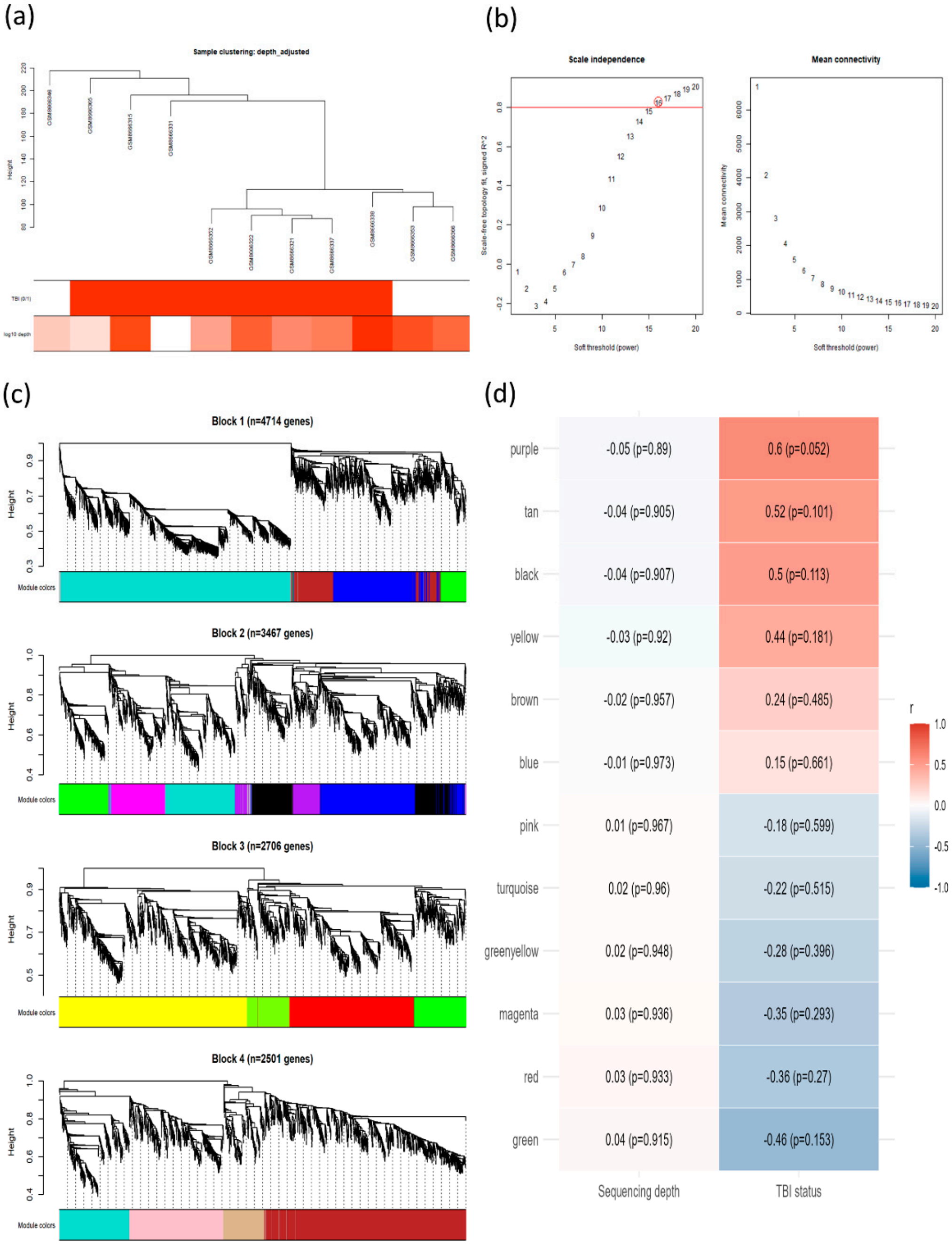

Figure S7. WGCNA of GSE283560 with 11 samples (7 dpi, depth adjusted). (a) Sample clustering dendrogram. (b) Soft-threshold selection; $\beta = 16$ ($R^2 = 0.825$) is marked. (c) Gene dendrogram of the 13,388 network genes in four blocks, with module colours (12 modules; 82 unassigned genes). (d) Module correlation (r, nominal p) with sequencing depth and TBI status; no module reached family-wise permutation $p < 0.05$ (Table S12).

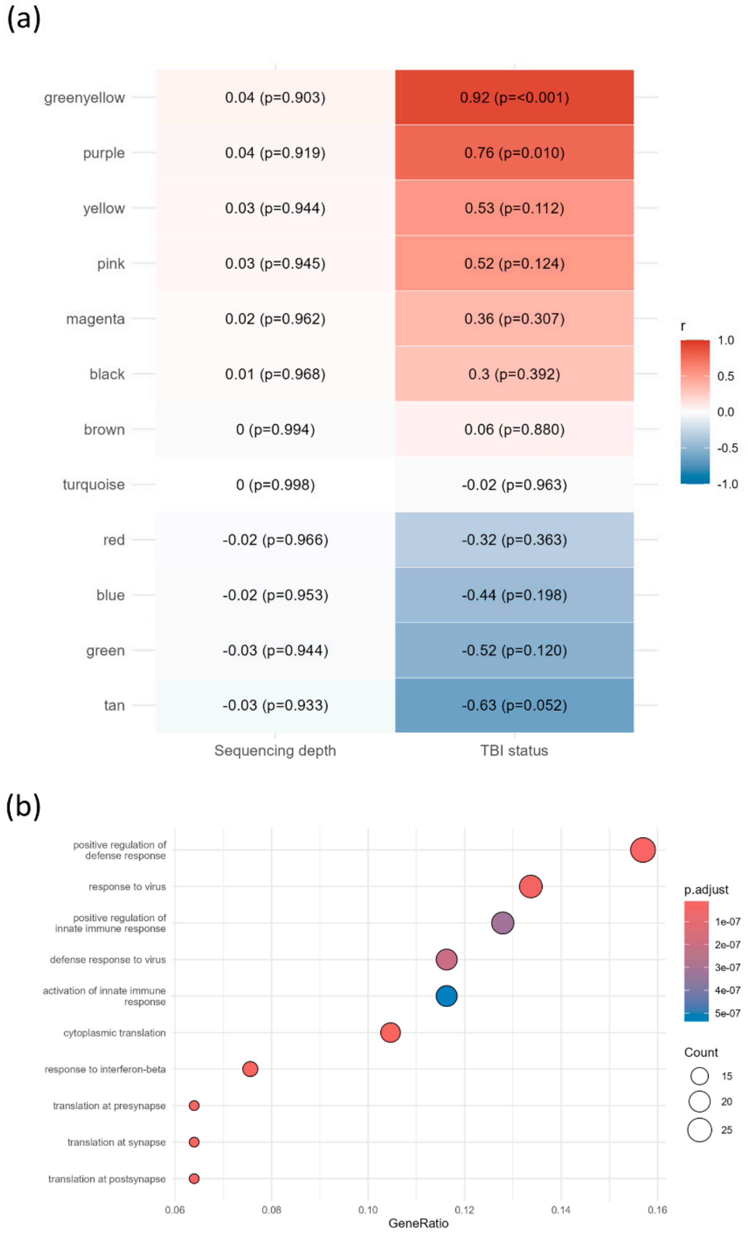

Figure S8. WGCNA after removal of GSM8666331 (10 samples). (a) Module correlation (r, nominal p) with sequencing depth and TBI status; the greenyellow module (210 genes) correlated with TBI ($r = 0.92$; family-wise permutation $p = 0.0083$, Table S15). $\beta = 18$ was used because no power reached $R^2 \geq 0.80$ ($R^2 = 0.53$). (b) GO Biological Process enrichment of the greenyellow module (Table S14). Module colours do not correspond to Figure S7. The result is exploratory (Table S15).

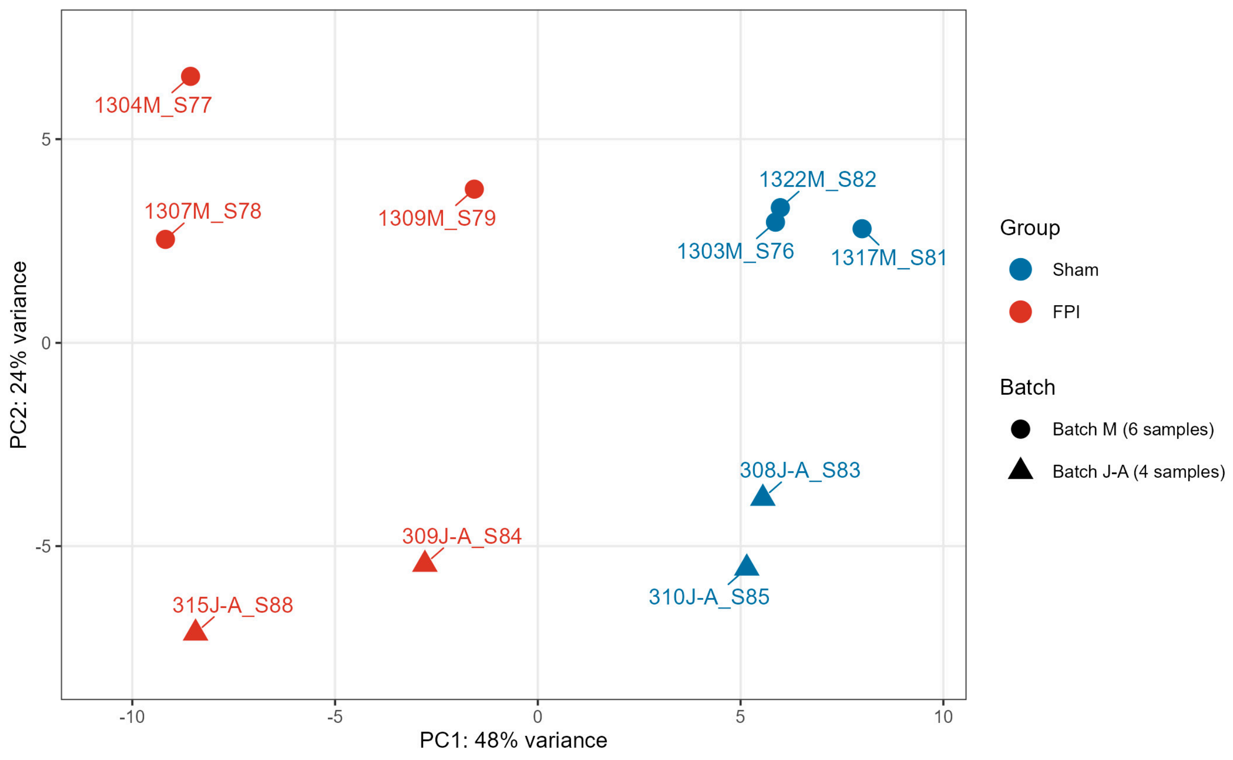

Figure S9. Principal component analysis of the ten GSE167459 microglia samples (variance-stabilized counts, top 500 genes by variance). Color shows injury group (Sham, FPI). Shape shows sequencing batch (circles: batch M, used in the primary analysis; triangles: batch J-A, the four samples not used in the primary analysis). Labels are sample codes. PC1 (48%) separates injury; PC2 (24%) separates batch.
